# Structure and Bonding in π-Stacked Perylenes: The Impact of Charge on Pancake Bonding

**DOI:** 10.1021/jacs.3c14065

**Published:** 2024-04-05

**Authors:** Rameswar Bhattacharjee, Henry Jervis, Megan E. McCormack, Marina A. Petrukhina, Miklos Kertesz

**Affiliations:** † Department of Chemistry and Institute of Soft Matter, Georgetown University, 37th and O Streets, NW, Washington, D.C. 20057-1227, United States; ‡ Department of Chemistry, University at Albany, State University of New York, 1400 Washington Avenue, Albany, New York 12222, United States

## Abstract

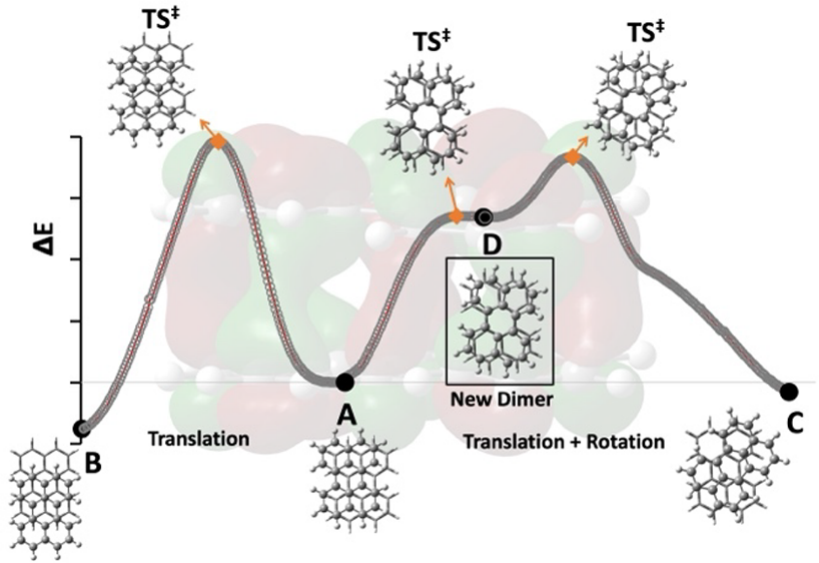

Perylene (PER) is a prototype of polycyclic aromatic hydrocarbons (PAHs), which play a pivotal role in various functional and electronic materials due to favorable molecule-to-molecule overlaps, which enhance electronic transport. This study provides guidelines regarding the impact of molecular charge on pancake bonding, a form of strong π-stacking interaction. Pancake bonding significantly boosts interaction energies within the monopositive dimer ([(C_20_H_12_)_2_]^•+^ or PER_2_^+^), crucial for stabilizing aggregation and crystal formation. We discovered energetically feasible sliding and rotation pathways within the [(C_20_H_12_)_2_]^•+^ dimer, connecting different configurations found in the Cambridge Structural Database (CSD). The dimer’s charge profoundly influences the pancake bond order (PBO) and the strength and structural preferences of pancake bonding. The most stable configuration is found in the monocationic state (PER_2_^+^), featuring a pancake bond order of 1/2 with one-electron multicenter bonding (1e/mc) with similar characteristics for charge −1. Increasing the total charge of the dimer to +2 or −2 leads to an unstable local minimum. Diverse distribution of pancake bonding types present in crystal structures is interpreted with modeling based on dimer computations with varying charges.

## 1 Introduction

Charge transfer salts of organic conjugated molecules exhibit diverse packing types, prominently featuring a rich variety of π–π stacking interactions,^1−3^ adopting various forms, ranging from monomeric to dimeric, and even extending to the formation of infinite π-stacks. The structural variations are attributed, in part, to the different charge states, which are characterized by the extent of charge transfer. The amount of charge transfer (CT) is influenced by several factors, as extensively discussed in the context of highly conducting CT salts like TTF-TCNQ.^1^^,^^2−5^ First, it stems from the difference between the ionization potential of the donor and the electron affinity of the acceptor. Second, the Coulomb interactions are strongly stabilizing as represented by the Madelung energy of the crystal.^6^ Additionally, delocalization energy, facilitated by intermolecular π–π overlap, plays a crucial role. These collective interactions under favorable circumstances contribute to the formation of metallic-like energy bands. These, plus dispersion interactions, ultimately determine the overall crystal packing.

In a specific group of intermolecular interactions involving polycyclic aromatic hydrocarbon (PAH) radicals, there exists a remarkable preference for π-stacking geometries often with shorter contact distances compared to van der Waals interaction.^3,7−9^ This intriguing phenomenon is referred to as “pancake bonding″, a concept originally proposed by Mulliken and Person.^10^ Pancake bond emerges when two singly occupied π-molecular orbitals (SOMOs) pair their spins, leading to intermolecular bonding interactions. This type of stabilization is referred to as an n-electron multicenter (ne/mc) covalent-like interaction.^11^ The specific π-stacking geometry is influenced by the shape of the SOMO.^8,12^ Recent progress in both experimental and computational studies has substantiated the widespread and robust nature of pancake bonding (PCB).^8,13−18^ These intermolecular interactions exhibit unique characteristics due to large direct overlap, notably the directional atom–over–atom π-stacking geometry. It is essential to maintain the highly overlapping π-stacking configuration for various applications involving intermolecular electron transfer.

Using advanced wave function quantum chemistry, Small et al. investigated the bonding energies of charged and neutral dimers of phenalenyl (PLY), a molecule known for its PCB behavior,^7^ and discovered that the PLY_2_^+^ cation radical dimer was bound by 20 kcal/mol while the neutral dimer was bound by only 11 kcal/mol. Despite the charged dimer having a formal pancake bond order (PBO) of 1/2 compared to 1 for the neutral dimer, the covalent contribution decreased and dispersion interactions remained largely unaffected. The significant increase in bonding energy was attributed to electrostatic effects. Cui and co-workers extended this study to charged dimers of olympicenyl (OPY) and perfluorinated PLY and OPY.^19^ Accordingly, the positively charged dimers exhibit stronger PCB than the neutral radical-based dimers. The larger interaction energy in charged pancake-bonded systems results partly from electrostatic effects in addition to the bonding interaction between SOMOs of the monomers.

**Scheme 1:**
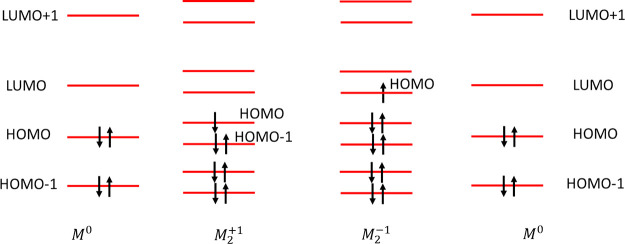
Orbital Occupancy of Ionic Pancake-Bonded Dimers of Molecule *M* For *M*_2_^+1^, the highest occupied molecular orbital, HOMO, and the one below, HOMO–1, of the dimer originate from the HOMO of the monomer. The HOMO is singly occupied and is antibonding between two monomers. HOMO–1 is doubly occupied and is bonding leading to an overall formal pancake bond order of PBO = 1/2. For *M*_2_^–1^, the HOMO orbital of the dimer originates from the lowest unoccupied molecular orbital, LUMO, of the monomer also with PBO = 1/2.

Perylene (to be referred to as (C_20_H_12_) or PER) is a prototypical closed-shell PAH. PCB is present in its salts, as our recent report^20^ showed for two crystal structures: [(C_20_H_12_)_2_]^•+^(SbCl_6_)^−^ from McCormack et al.^20^ and [(C_20_H_12_)_2_]^•+^(C_32_H_12_BF_24_)^−^ from Rosokha et al.^21^ PCB in both salts arises from the balance of the bonding interaction in the HOMO–1 of the dimer occupied by two electrons and the antibonding interaction in the HOMO orbital of the dimer occupied by only one electron producing PBO of 1/2, as illustrated in Scheme 1.

Inspired by the seminal works on PLY dimers,^3,7,19^ we set to systematically explore the effects of the charge per perylene on the intermolecular interactions in their dimers. We analyzed the Cambridge Structural Database (CSD) and found that the CSD^22^ has 24 experimental crystal structures containing π-stacking perylenes, which are all cationic. These systems feature at least one first-neighbor perylene π-stacking pattern. In addition, the CSD contains nine anionic PER crystal structures that are listed in Table S6 in the SI section. These crystals, such as HEDXUJ^23^ (C_16_H_36_NaO_8_^+^, C_20_H_12_^–^, C_20_H_12_), contain PER anions, but none displays PCB. This is a puzzling fact, and this work provides theoretical explanation for the lack of PCB in the anionic perylenes in contrast to the cationic dimers.

For the cationic perylenes, the charges assignable to each perylene vary in a wide range from *Q* = 0.0 to 1.0 per monomer, as will be discussed below. In fact, one of the challenges of this investigation is to obtain a charge assignment in each experimental perylene structure. This will be accomplished by relying on a correlation between the monomer charges and their bond length alternation parameter, BLA, as defined in the Methods section. We used dimers with varying charges without the counterions to model structural motifs in the crystals. This is a rather strong approximation, given the large Coulomb interaction between anions and cations. The reason this model has been successful to a good degree is that PCB is directional while the Coulomb interaction is less so. Therefore, the model assumes indirectly that the structural motifs are largely governed by the directional component of the total energy. This approach has a history of success for other pancake-bonded CT salts, as for example, 252 salts of TCNQ with π-stacking geometry have been analyzed in this manner, finding that the pancake-bonded maximum overlapping configurations are dominant in the distribution of the first-neighbor TCNQ anions whether fully or only partially charged.^24^

While the presented modeling is done on dimers, the experiments do not refer to very well-isolated dimers except for WIKZOF, (C_20_H_12_^+^, C_20_H_12_, F_6_P^–^), and CUWBIF, (C_20_H_12_^+^, 5(C_20_H_12_), ClO_4_^–^); the rest of the structures are more complex. Therefore, when analyzing the X-ray diffraction (XRD) structures, and describing the PER-to-PER stacking, we will use the term “π-stacking motif” as shorthand for “first neighbor perylene π-stacking dimeric pattern”.

We modeled perylene dimer units by referring to the distinct experimentally observed π-stacking structures from the literature. Specifically, we designated the perylene dimer derived from or resembling the structure in XIWQON, ([(C_20_H_12_)_2_]^•+^,(SbCl_6_)^−^),^20^ as “Type A”, the dimer derived from or resembling the structure in DIQDEO, ([(C_20_H_12_)_2_]^•+^,(C_32_H_12_BF_24_)^−^),^21^ as “Type B”, and the parallel-rotated dimer based on or resembling the structure in ECINAF, (2(C_20_H_12_^+^), 3(C_20_H_12_), Mo_6_O_19_^2–^),^25^ as “Type C”. “Type F” refers to the fully overlapping configuration.

We are particularly interested in understanding four aspects:

1. The bonding interactions within each of the four types of perylene dimers.
2. Investigating transitions and barriers under sliding and rotation among different dimers.
3. Evaluating the effects of overall charge on the intermolecular pancake bond between two perylene molecules in dimer models.
4. Interpreting the experimental crystal structures and the distribution of the charges among the different symmetry unrelated perylenes in them.

The rest of the article is structured as follows:

Computational methods
Overview of available crystal structures
Results of dimer computations as a function of dimer charge, *q*, and their comparisons
Discussion of crystal structures for *q* = +1, where most of the experimental data are available
Comments on spectra, and conclusions.

## 2 Computational Details

To gain insights into the interactions between two PER monomers in dimeric structures, we employed density functional theory (DFT) calculations. Geometry optimizations were performed using the (U)M05-2X^26^/6-311G(d) level of theory, where the spin-unrestricted (U) formalism was utilized for open-shell systems. This choice DFT functional is based on a previous in-depth analysis of four pancake-bonded π-dimers, which demonstrated that M05-2X outperformed more than 50 other modern DFT functionals (many including additional D3 dispersion terms)^27^ when compared to the highly accurate multi reference average quadratic coupled cluster MR-AQCC method.^28^ This success is partly due to the fact that M05-2X incorporates certain dispersion effects.^29^ We also examined the influence of integrating additional D3 dispersion^30^ corrections on our findings, focusing on their effects on binding energies and optimized geometries. The inclusion of D3 corrections typically resulted in significantly larger (more negative) interaction energies, with an average increase of about 6.0 kcal/mol.

As outlined in the introduction, different total charges (*q*) on the dimers were considered. *Q* refers to the formal charge per monomer. All stationary points were confirmed by the absence of imaginary frequencies, and the transition structures (TS) were verified by the presence of a single imaginary frequency. In the optimization of these TS structures, we initialized the calculations with starting guesses obtained from QST2 calculations.^31^ Additionally, we validated that each computed TS connects the correct reactant and product on the potential energy surface (PES) corresponding to a given dimer charge, *q*, by conducting intrinsic reaction coordinate (IRC) calculations. The calculations were carried out using the Gaussian 16 program.^32^ CHELPG (Charges from Electrostatic Potentials using a Grid-based method) was utilized for the calculation of atomic charges as implemented in Gaussian.^33^ The isovalue of 0.015 was used throughout. Hückel level orbitals were obtained by the HuLiS program.^34^

Bond length alternation (BLA) values correlate with molecular charge in conjugated molecules.^35^ BLA is defined along a particular conjugation path:
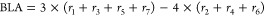
1

**Scheme 2:**
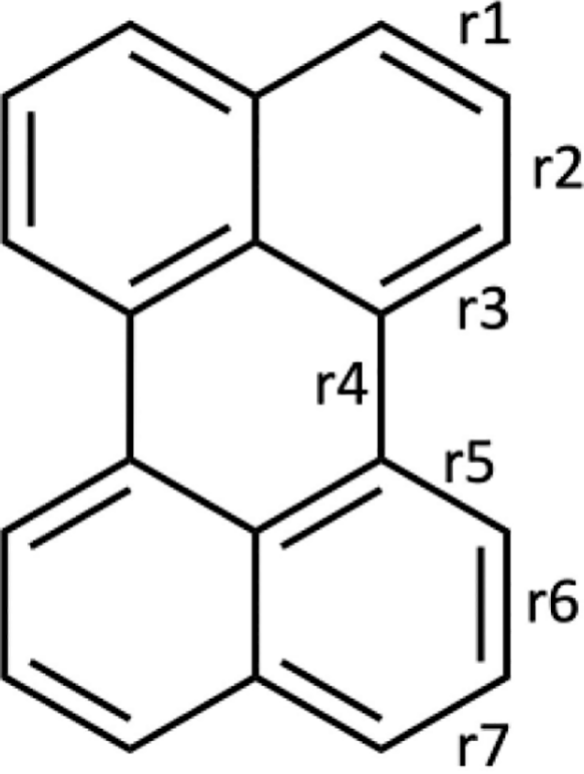
Bond Lengths Used to Calculate Bond Length Alternation (BLA)

The bond numbering follows Scheme 2. In case there is no symmetry in the XRD structure, an average is taken.

The interaction energy is defined as (*X* refers to the dimer type):
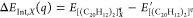
2Here, *E*_[(**C20H12**)**2**]^*q*^_^′^ denotes the energy of the dimer with a total charge of *q* when the monomers are separated sufficiently far to cancel intermolecular interactions; the corresponding parallel displaced distance is at least 20 Å. *E* refers to the optimized structure of the dimer with the same total charge.

## 3 Results and Discussion

### 3.1 Crystal Structures of Perylene Salts

**Scheme 3:**
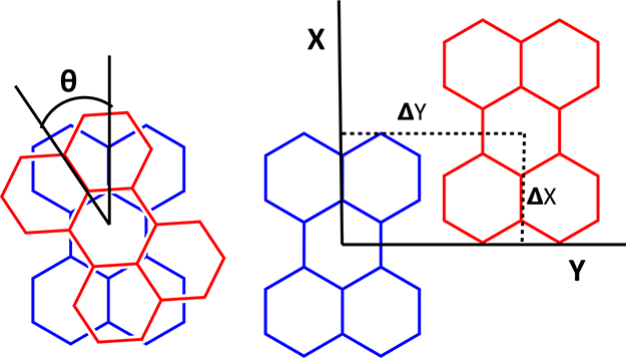
Definition of the Rotation and Translation Parameters (θ, **Δ***X*, and **Δ***Y*) Used to Characterize the Relative Molecular Geometry in the π-Stacking Perylene Dimer **Δ***Z* is the parallel displacement parameter of the order of 3.2 to 3.5 Å in the discussed geometries.

**Figure 1:**
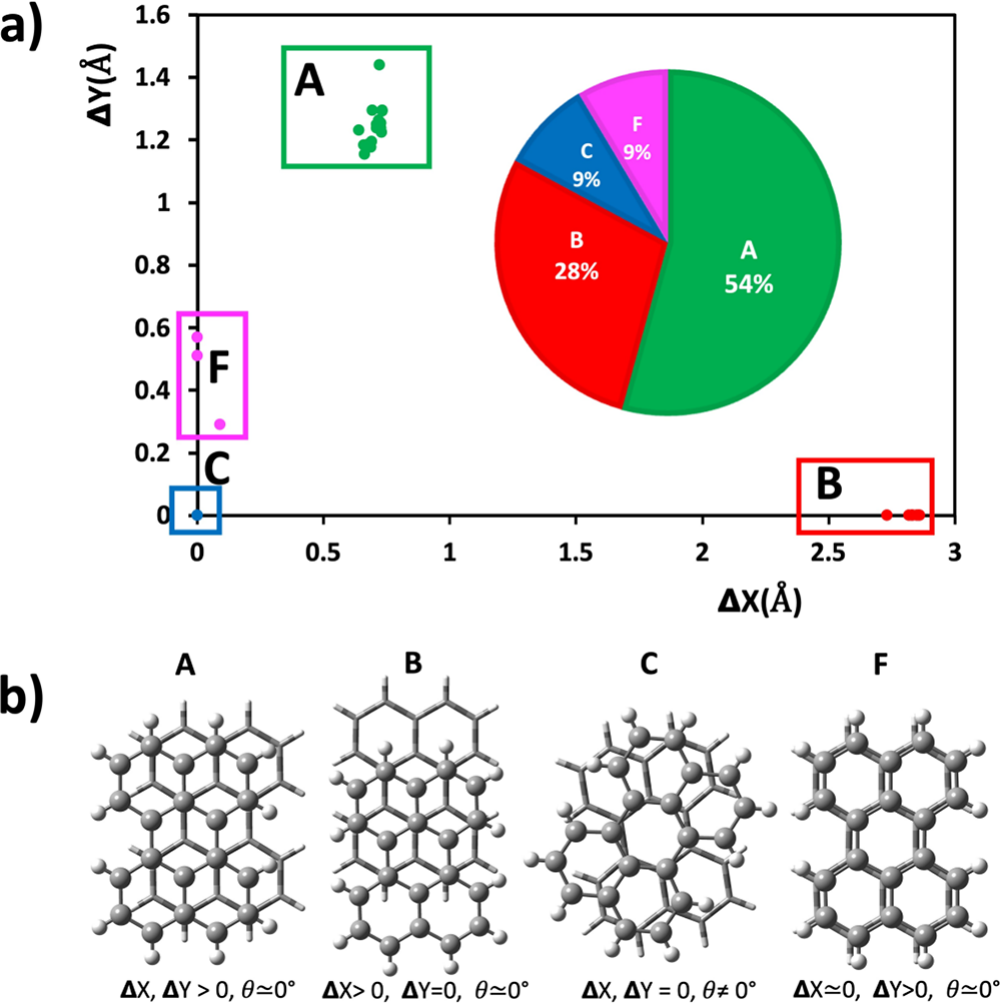
(a) Distribution of Δ*X* and Δ*Y* values are based on crystal structures of perylene salts with motifs tabulated in Table 1 from the CSD. The inset in Figure 1a shows the distribution and prevalence of the four π-stacking motifs in the literature. Regions in green, red, and blue refer to structures of type A, type B, type C, and type F, respectively. (b) The four unique π-stacking motifs found in the CSD. θ, Δ*X*, and Δ*Y* refer to Scheme 3. Typical observed values for type C are close to θ = 40°. Type D (not shown here) is very similar to type F and is found only in the computations.

As shown in Figure 1, the CSD contains four distinct π-stacking perylene first-neighbor π-stacking motifs. These are classified based on the relative orientation of the perylene molecules using three parameters, θ, Δ*X*, and Δ*Y*, as illustrated in Scheme 3.

Table 1 lists key parameters of crystal structures associated with π-stacking motifs found in perylene salts from the CSD limited the *R*-factors <6%. Table S4 in the SI lists all 24 structures.

**Table 1:** Perylene Structural Analysis Indicating Packing Parameters of the Shortest Pancake Bonding (PCB)^a^

CSD ref code, reference	chemical formula	contacts^b^	PCB parameters^c^(distances in Å, angles in degrees)	*R*-factor
XIWQON^20^	[(C_20_H_12_)_2_]^•+^(SbCl_6_)^−^	A (8)	θ = 0.24, **Δ***X* = 0.699, **Δ***Y* = 1.230, **Δ***Z*_Avg_ = 3.309	*R* = 3.04%
WERVAR^36^	2(C_20_H_12_^+^), C_20_H_12_, 2(C_8_AuF_6_N_2_S_4_^–^)	A (2)	θ = 0.53, **Δ***X* = 0.723, **Δ***Y* = 1.246, **Δ***Z*_Avg_ = 3.417	*R* = 2.58%
RUVGIY^37^	C_20_H_12_^+^, C_20_H_12_, C_16_H_8_CuN_4_S_4_^–^	A (2)	θ = 0.34, **Δ***X* = 0.726, **Δ***Y* = 1.239, **Δ***Z*_Avg_ = 3.436	*R* = 5.49%
ECINEJ^25^	3(C_20_H_12_^+^), 2(C_20_H_12_), O_19_VW_5_^3–^	F (20)C (1)A (2)	C: θ = 41.31, **Δ***X* = 0, **Δ***Y* = 0, **Δ***Z*_O_ = 3.433 A: θ = 0.41, **Δ***X* = 0.727, **Δ***Y* = 1.253, **Δ***Z*_Avg_ = 3.481	*R* = 5.62%
DIQDEO^21^	C_20_H_12_^+^, C_20_H_12_, C_32_H_12_BF_24_^–^	B (7)	θ = 1.53, **Δ***X* = 2.831, **Δ***Y* = 0, **Δ***Z*_Avg_ = 3.322	*R* = 5.55%
ECINAF^25^	2(C_20_H_12_^+^), 3(C_20_H_12_), Mo_6_O_19_^2–^	C (5)A (2)F (5)	C: θ = 42.56, **Δ***X* = 0, **Δ***Y* = 0, **Δ***Z*_O_ = 3.430 A: θ = 0.10, **Δ***X* = 0.709, **Δ***Y* = 1.238, **Δ***Z*_Avg_ = 3.506	*R* = 3.5%
PAJXAZ01^38^	C_20_H_12_^+^, C_20_H_12_, C_8_CoN_4_S_4_^–^	B (6)	θ = 0.04, **Δ***X* = 2.850, **Δ***Y* = 0, **Δ***Z*_Avg_ = 3.353	*R* = 4.4%
RIMBOH^39^	C_20_H_12_^+^, C_20_H_12_, C_8_N_4_PtS_4_^–^	B (7)	θ = 0.14, **Δ***X* = 2.817, **Δ***Y* = 0.0, **Δ***Z*_Avg_ = 3.30	*R* = 1.91%
WIKZOF^40^	C_20_H_12_^+^, C_20_H_12_, F_6_P^–^	A (4)	θ = 1.12, **Δ***X* = 0.687, **Δ***Y* = 1.176, **Δ***Z*_Avg_ = 3.396	*R* = 5.86%

a Only structures with *R* < 6% are shown. The full list of 24 structures without this restriction is in Table S4 in the SI section.

b Contact type and number of short C···C contacts.

c PCB parameters of the first neighbor perylene π-stacking θ, **Δ***X*, **Δ***Y*, and **Δ***Z*. Only the shortest is shown if several are present. **Δ***Z*_Avg_ is the average over the short C···C stacking contacts, **Δ***Z*_O_ is the center-to-center distance in the C-type structures.

### 3.2 Dimer Models as a Function of Charge

Table 2 summarizes key information on the optimized dimers of perylene as a function of overall charge of the dimer, *q*. Various minima were obtained, and they are identified by the letter code used in Figure 1 as types A, B, and C. In addition, a new π-stacking structure was identified as dimer type D (more on type D; see Figure 5 and Figure S4). Note that the fully overlapping structure (F in Figure 1) is not a local minimum for any of the five dimer charges.

**Table 2:** Computed Data on Pancake Bond Formation within Perylene Dimers across Different Types and Charge States Using Full Geometry Optimization with (U)M05-2*X*/6-311G(d)

optimized dimer [(C_20_H_12_)_2_]*_X_^q^*	charge (*q*)^a^	charge distribution	optimized **Δ***X*, **Δ*Y***, **Δ*Z,*** and θ, values	*E*_Int,*X*_(*q*) (kcal/mol)	no. of short C···C	pancake interaction
[(C_20_H_12_)_2_]*_A_^q^*	+2	[(PER)^+1.0^(PER)^+1.0^]	0.46, 1.25, 3.38, 0.85°	+18.7	8	Present
[(C_20_H_12_)_2_]*_B_^q^*	+2	[(PER)^+1.0^(PER)^+1.0^]	2.57, 0.0, 3.30, 0.0°	+17.3	7	Present
[(C_20_H_12_)_2_]*_C_^q^*	+2	[(PER)^+1.0^(PER)^+1.0^]	0.41, 1.13, 3.40, 35.6°	+18.8	8	Present
[(C_20_H_12_)_2_]*_A_^q^*	+1	[(PER)^+0.5^(PER)^+0.5^]	0.31, 1.15, 3.27, 0.51°	–19.6	8	Present
[(C_20_H_12_)_2_]*_B_^q^*	+1	[(PER)^+0.5^(PER)^+0.5^]	2.53, 0.0, 3.29, 0.02°	–20.4	7	Present
[(C_20_H_12_)_2_]*_C_^q^*	+1	[(PER)^+0.5^(PER)^+0.5^]	0.0, 0.0, 3.25, 41.7°	–19.9	8	Present
[(C_20_H_12_)_2_]*_D_^q^*	+1	[(PER)^+0.5^(PER)^+0.5^]	0.45, 0.0, 3.41, 12.5°	–16.9	7	Weak^b^
[TS]_1_^•+^	+1	[(PER)^+0.82^(PER)^+0.18^]	1.48, 1.06, 3.37, 0.52°	–15.7	9	Absent
[TS]_2_^•+^	+1	[(PER)^+0.5^(PER)^+0.5^]	0.47, 0.25, 3.36, 11.2°	–16.9	6	Weak
[TS]_3_^•+^	+1	[(PER)^+0.81^(PER)^+0.19^]	0.72, 0.0, 3.35, 21.6°	–16.0	6	Absent
[(C_20_H_12_)_2_]*_A_^q^*	0	NA	1.01, 1.24, 3.34, 0.0°	–13.5	-	vdW^c^
[(C_20_H_12_)_2_]*_B_^q^*	0	NA	2.85, 0.0, 3.35, 0.41°	–11.1	-	vdW
[(C_20_H_12_)_2_]*_C_^q^*	0	NA	0.0, 0.0, 3.31, 32.0°	–13.6	-	vdW
[(C_20_H_12_)_2_]*_A_^q^*	–1	[(PER)^−0.5^(PER)^−0.5^]	1.31, 0.0, 3.28, 0.05°	–12.5	8	Present
[(C_20_H_12_)_2_]*_B_^q^*	–1	[(PER)^−0.5^(PER)^−0.5^]	3.05, 0.0, 3.21, 0.0°	–13.9	7	Present
[(C_20_H_12_)_2_]*_C_^q^*	–1	[(PER)^−0.5^(PER)^−0.5^]	0.0, 0.0, 3.37, 33.4°	–9.1	6	Present
[(C_20_H_12_)_2_]*_B_^q^*	–2	[(PER)^−1.0^(PER)^−1.0^]	3.21, 0.0, 3.28, 0.11°	+26.4	7	Present

a Total charge per dimer.

b Weak pancake bonding; see text.

c vdW indicates a van der Waals dimer stabilized by dispersion.

As is evident from Table 2, the overall charge of the dimer plays a crucial role in shaping its electronic properties and its structures. Since most experimental data refer to the *q* = +1 charge, these are discussed first, including transitions among the local minima on the *q* = +1 PES, followed by the other *q* values in turn. After the discussion of the computational results, various observed XRD structures from the literature will be analyzed based on the computational results leading to a broader understanding of PCB.

**Figure 2:**
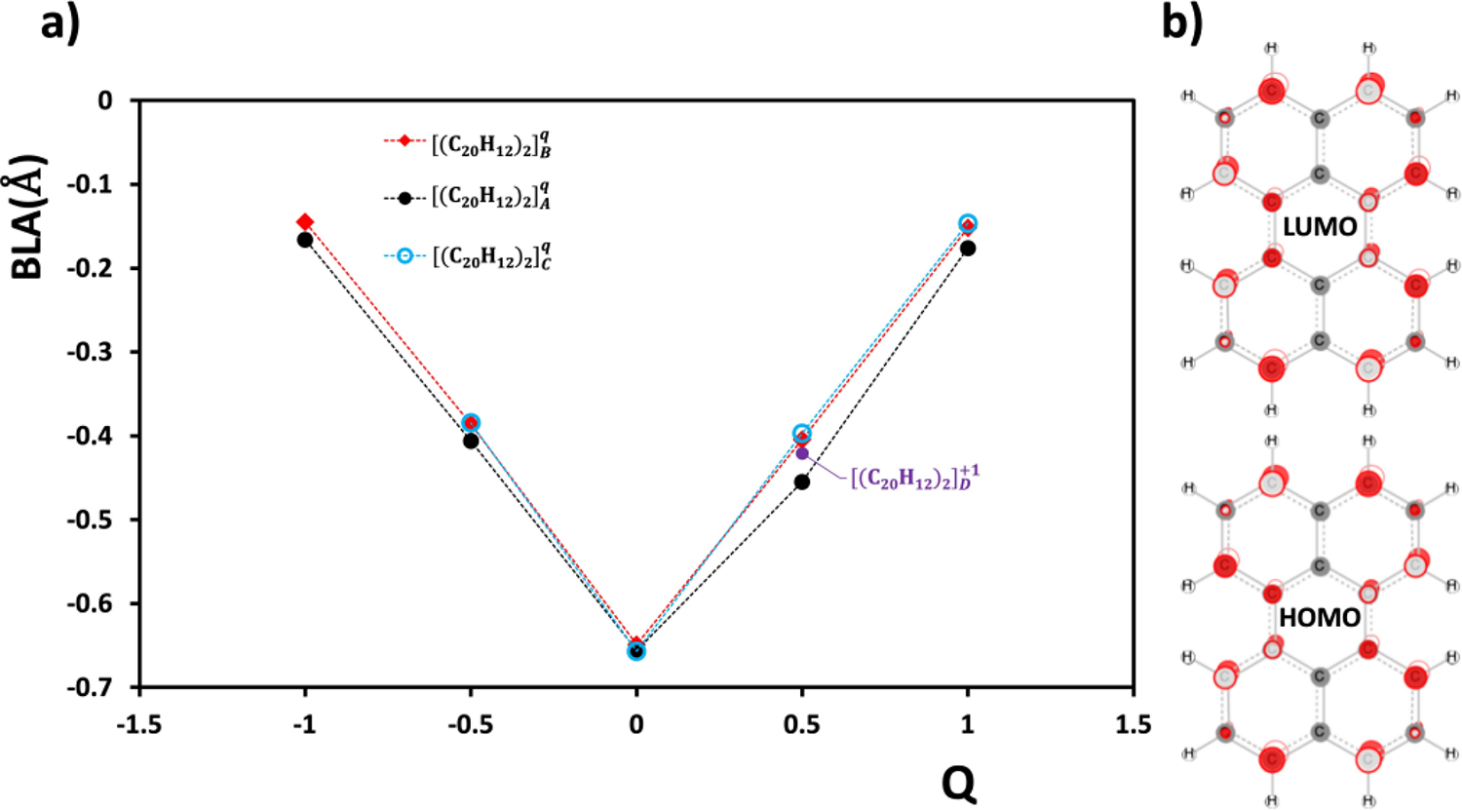
(a) Computed BLA values, defined in eq 1, are shown as a function monomer charge (*Q*) for differently charged dimers of perylene at their respective optimized geometries. The total charge of the dimer is *q* = 2*Q*. Lines are provided to guide the eye. The charge distribution between the two perylenes is even in all cases. (b) Hückel level HOMO and LUMO of the neutral perylene monomer.

Figure 2a shows the correlation between the DFT computed BLA values and the charge per monomer. Three different computations are shown, all closely following a V-shaped trend resulting from the bonding/antibonding characters of the orbitals shown in Figure 2b. The minor differences in the BLA values for each *Q* value are due to small differences in the packing of the monomers in each dimer. This correlation will serve as the basis for assigning molecular charges for the crystal structures under discussion.

### 3.3 Perylene Dimers with Overall Charge of *q* = +1, Computations

**Figure 3:**
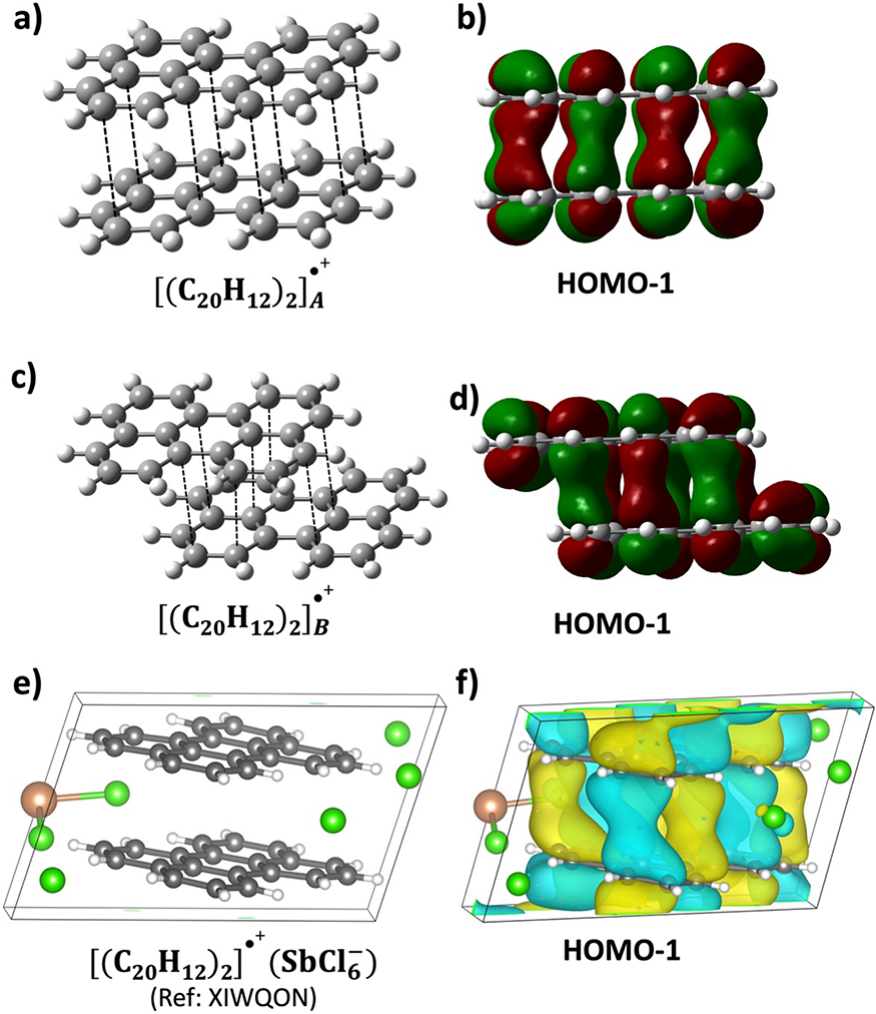
Optimized structures and orbitals, highlighting pancake bonding interactions within monocationic perylene dimers (*q* = +1). (a) [(C_20_H_12_)_2_]_A_^•+^. (b) Orbital corresponding to [(C_20_H_12_)_2_]_A_^•+^. (c) [(C_20_H_12_)_2_]_B_^•+^. (d) Orbital corresponding to [(C_20_H_12_)_2_]_B_^•+^. (e) Unit cell of XIWQON [(C_20_H_12_)_2_]^•+^(SbCl_6_)^−^. (f) HOMO–1 orbital of the XIWQON unit cell. Partly adapted with permission from ref (20). Copyright 2023 American Chemical Society.

**Figure 4:**
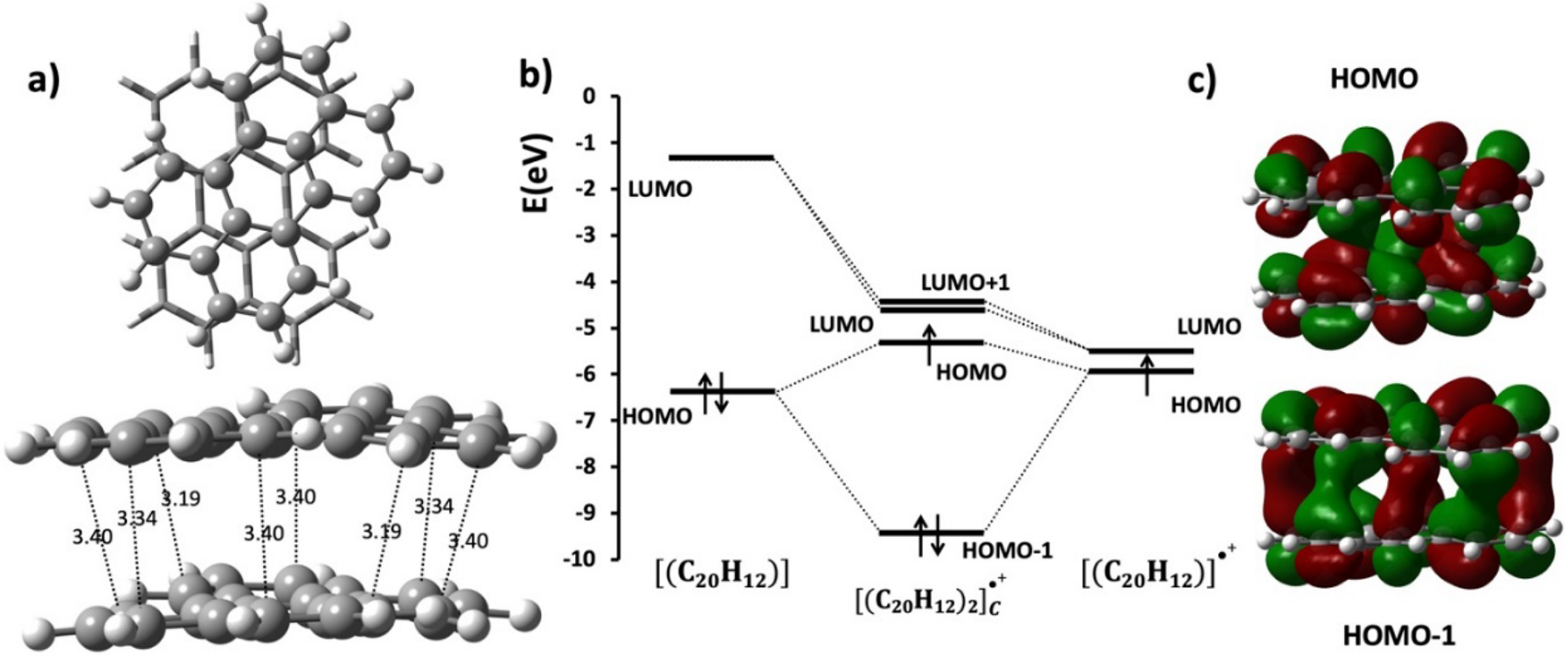
(a) Top and side views of the optimized structure of [(C_20_H_12_)_2_]_C_^•+^ with intermolecular C···C short contacts provided in Å. (b) The molecular orbital diagram of the [(C_20_H_12_)_2_]_C_^•+^. (c) HOMO–1 and HOMO orbital of [(C_20_H_12_)_2_]_C_^•+^.

The interaction energies of the four minima at *q* = +1 are listed in Table 2 and range from −16.6 to −20.4 kcal/mol. These are substantial interaction energies considering the repulsive electrostatic energy expected to arise from the +0.5 charge on each monomer in considerable vicinity of each other. This indicates PCB, as detailed below with PBO = 1/2, as illustrated in Scheme 1. All four structures display different geometries with different short C···C contacts and different HOMO–1 orbitals, which are the lead orbitals in driving PCB as per Scheme 1. These variations were quantified by the optimized **Δ***X* and **Δ***Y* values associated with each dimer. Specifically, the dimers denoted as [(C_20_H_12_)_2_]_A_^•+^and [(C_20_H_12_)_2_]_C_^•+^ contain eight short contacts, while the third type of dimer, [(C_20_H_12_)_2_]_B_^•+^, possesses seven short contacts shown in Figures 3 and 4. We have identified a strong PCB interaction in these three [(C_20_H_12_)_2_]^•+^ dimers with short **Δ***Z* values, except for the D-type structure to be discussed below.^20^ It is an important outcome that these dimers display relatively large interaction energies between the perylene molecules, close to the value found for the PLY_2_^+^ cation radical dimer of −20 kcal/mol.^7,19^ The interaction energies around −20.0 kcal/mol are directly related to PCB in the dimers, which is further confirmed by the direct C···C intermolecular orbital overlaps seen in the HOMO–1 in Figures 3 and 4. The bonding characteristics of the HOMO–1 orbital in [(C_20_H_12_)_2_]^+^ have been further corroborated through calculations using periodic boundary conditions on the crystal [(C_20_H_12_)_2_]^•+^(SbCl_6_)^−^ (ref XIWQON). This compound, featuring an isolated perylene dimer carrying a total formal charge of +1, offers an exemplary basis for comparison with isolated dimers. As depicted in Figure 3f, the HOMO–1 orbital exhibits intermolecular orbital overlap similar to the molecular model in Figure 3b, indicating its key role in the formation of the pancake bond. (For additional details on the periodic computations, see the Supporting Information.)

For both A and B types, the orientation of the optimized perylene molecules is aligned in the same direction (θ ≈ 0°) while the C-type dimers are oriented at an angle of θ ≈ 40°, similar to the crystal structures.^25^ The relative rotation allows the appearance of different short contacts, as observed in the optimized geometry shown in Figure 4a. The molecular orbital diagram, shown in Figure 4b for [(C_20_H_12_)_2_]_C_^•+^, is also indicative of PCB with PBO = 1/2.

The primary distinction between the dimeric structures of [(C_20_H_12_)_2_]_A_^•+^, [(C_20_H_12_)_2_]_B_^•+^, and [(C_20_H_12_)_2_]_C_^•+^ lies in how the monomers are arranged relative to each other. Since these dimer configurations represent stable local minima on the PES, it is possible to transform one dimer into another by sliding and/or rotation. Such a transition between different minima requires overcoming an energy barrier and passing through a TS.

**Figure 5:**
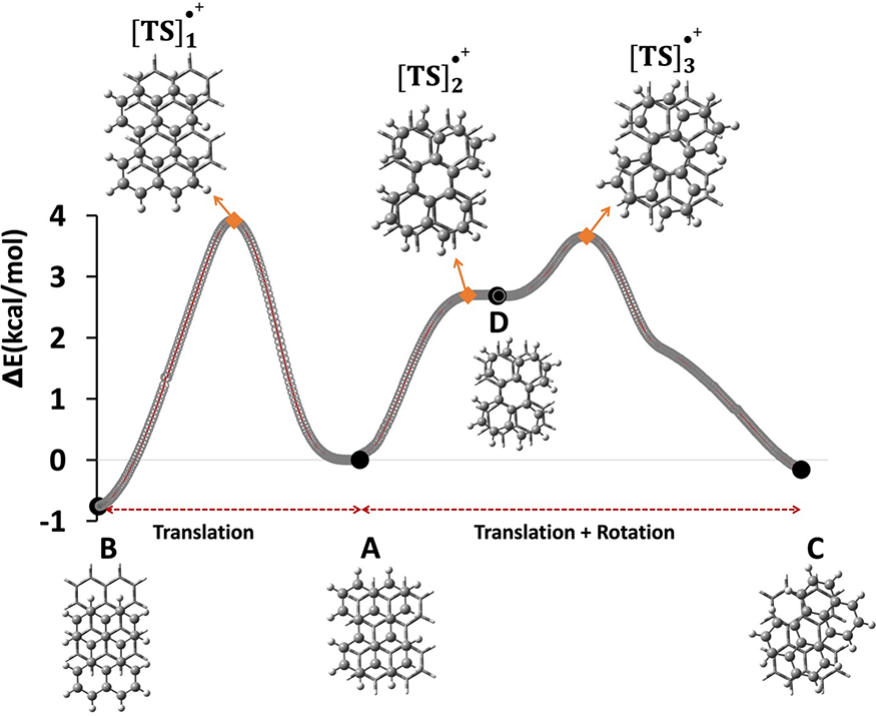
Energy diagram of the conversion of [(C_20_H_12_)_2_]_A_^•+^ to [(C_20_H_12_)_2_]_B_^•+^ and to [(C_20_H_12_)_2_]_C_^•+^. Solid black points correspond to the local minima on the PES whereas diamond orange markers denote transition structures (TSs). The full reaction path in Figure S5 incorporates all three parameters (Δ*X*, Δ*Y*, and θ) together.

We identified the TSs involved in the transformation between different dimers and computed the PES along these transformations illustrated in Figure 5. The TS involved in the transformation from the A to B types is [TS]_1_^•+^ and is 3.9 kcal/mol above [(C_20_H_12_)_2_]_A_^•+^ and 4.7 kcal/mol above [(C_20_H_12_)_2_]_B_^•+^, respectively. [TS]_2_^•+^ was confirmed by IRC calculations connecting [(C_20_H_12_)_2_]_A_^•+^ on the reactant side. However, moving forward toward the “C” side, an unexpected minimum is found (D-type), denoted as [(C_20_H_12_)_2_]_D_^•+^. The stability of the [(C_20_H_12_)_2_]_D_^•+^ structure was also confirmed by the absence of any imaginary vibrational frequency. The relevant orbital shows PCB in [(C_20_H_12_)_2_]_D_^•+^, characterized by eight C···C short contacts (see Figure S4 for details). By locating [TS]_3_^•+^, we computed the activation energy for the conversion from [(C_20_H_12_)_2_]_D_^•+^ to [(C_20_H_12_)_2_]_C_^•+^, as 1 kcal/mol.

**Figure 6:**
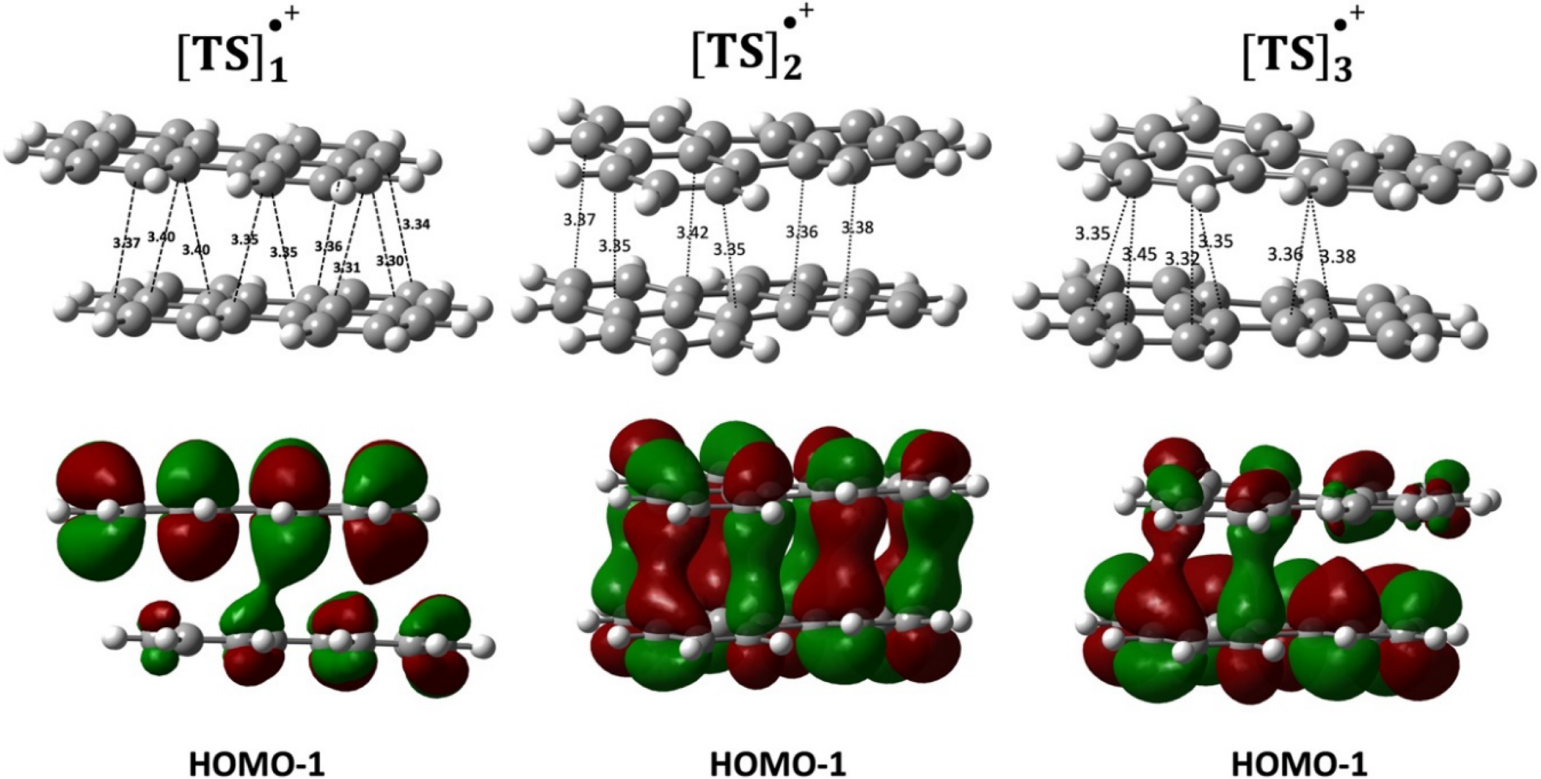
Side view of the three optimized transition structures shown in Figure 5 with the C···C short contacts given in Å. The HOMO–1 orbital below each structure shows the persistence of pancake bonding interactions in [TS]_2_^•+^ but not in the other two.

The optimized structure of [TS]_1_^•+^ is depicted in Figure 6, and it demonstrates no direct atom–over–atom contacts. However, the HOMO–1 orbital shows intermonomer overlap that is barely reminiscent of the pancake bond in [TS]_1_^•+^. Additionally, the spin densities (see Figure S6) show that the spin is highly localized on one of the monomers in [TS]_1_^•+^, in agreement with a CHelpG charge analysis.^33^ The charge distribution in [TS]_1_^•+^ is uneven as indicated in Table 2 with an assignment of [(C_20_H_12_)^+0.82^(C_20_H_12_)^+0.18^]. This is another sign of the absence of significant PCB in [TS]_1_^•+^ and similarly in [TS]_3_^•+^.

The accurate computation of the strength of PCB is hampered by finding a proper reference interaction energy, unlike in the case of the PLY dimer, where at a rotational angle of 30°, PCB disappears due to symmetry.^9^ At this TS, the spins become localized on the SOMOs of the monomers due to the reduced intermolecular overlap while the remainder of the interaction energy can be approximately attributed to the bare vdW interaction, without pancake interaction.

Such a localization of the unpaired electron occurs in [TS]_1_^•+^ as mentioned above and also in [TS]_3_^•+^ but not in [TS]_2_^•+^. Based on the interaction energy values for *q* = 0 ranging from −11.1 to −13.6 kcal/mol, we approximate the underlying vdW interaction energy at −12.3 ± 1 kcal/mol. Then, the pancake interaction energy for [(C_20_H_12_)_2_]_A_^•+^ is approximately ≈ −7.3 kcal/mol. A similar estimate for [(C_20_H_12_)_2_]_B_^•+^ and [(C_20_H_12_)_2_]_C_^•+^ would yield ≈−7.8 kcal/mol and ≈−7.6 kcal/mol, respectively. By the same argument, [(C_20_H_12_)_2_]_D_^•+^ can be described as having only weak PCB in the order of ≈−4.6 kcal/mol and the nearby [TS]_2_^•+^ retains also nearly the same strength of PCB.

### 3.4 Perylene Dimers with Overall Charge of *q* = 0: Computations and Experiments

For completeness, we mention that the crystal packing in neutral perylene, PERLEN05,^41^ being a closed shell molecule, displays a herringbone arrangement of dimers. The dimers themselves show a graphite-like AB packing with atoms over near centroids of six-membered rings. This is a typical vdW packing, without any sign of PCB. The first-neighbor packing in this case corresponds to **Δ***X* = 1.2, **Δ***Y* = 0.7, θ = 0, close to the respective values for [(C_20_H_12_)_2_]_*A*_^0^ in Table 2. The fact that there is only a qualitative agreement with the computed dimer minimum and that there are two more minima in Table 2 reflects a typical situation with vdW intermolecular interactions where there are multiple energetically close-lying minima.

### 3.5 Perylene Dimers with Overall Charge of *q* = −2, −1, and +2: Computations

Next, we discuss how the overall charge affects the structures and electronic properties of the [(C_20_H_12_)_2_]^*q*^ dimers. Given that the driving force for *q* = +1 and +2 relies on the same orbital (the HOMO–1 of the dimer, as per Scheme 1), one expects a close structural correlation between the local minima between *q* = +1 and +2. Indeed, the pairwise structures for [(C_20_H_12_)_2_]_*A*_^+1^ and [(C_20_H_12_)_2_]_*A*_^+2^ are nearly identical. The similarity between the minima for [(C_20_H_12_)_2_]_B_^+1^ and [(C_20_H_12_)_2_]_B_^+2^ is also striking. For the C-type structures between *q* = +1 and +2, there is a significant difference. While the degrees of rotations are very similar, the Δ*Y* values of the local minima differ by 1.13 Å. However, the interaction energies for *q* = +2 are all positive, indicating that these local minima on the dimer PES would not be stable without the counterions. In fact, no experimental structure with *q* = +2 has been found so far.

The situation with the *q* = −1 and −2 pair is different compared to the respective positive counterparts. The main reason for this difference is that the driving force for PCB in this case is due to the stabilization of the HOMO of the dimer, which is derived from the LUMOs of the monomers as indicated by Scheme 1. While the two minima [(C_20_H_12_)_2_]_B_^–1^ and [(C_20_H_12_)_2_]_B_^–2^ correspond to two nearly identical structures, for *q* = −2, the A- and C-type local minima do not even exist. Another even more significant difference is due to the charge distribution of the π–electron cloud over the PAHs that lends an electrostatic repulsion element to the face-to-face π–stacking of neutral PAHs. This repulsion is reduced for *q* > 0 but enhanced for *q* < 0. This circumstance is behind the fact that the sole local minimum on the *q* = −2 surface is overall highly repulsive and that the three local minima on the *q* = −1 surface are much less attractive than the respective *q* = +1 and +2 cases. The salts of perylene anions listed in Table S6 show only isolated perylenes and not π-stacking perylenes, a fact in line with the overall smaller interaction energy for anion dimers of perylene.

To evaluate the stability of the dimers, we also determined the free energy of interaction (*G*_Int_ at 298.15 K) between perylene molecules at room temperature. This calculation includes the energy due to entropy loss associated with dimerization. *G*_Int_ values are negative for all examined dimers, suggesting that their formation is thermodynamically favorable under standard temperature conditions. The entropy loss has been calculated to range from 17.0 to 38.0 cal·mol^–1^·K^–1^ for neutral, monocationic (*q* = +1), and monoanionic (*q* = −1) dimers. We provide the zero-point energy (ZPE)-corrected interaction energy (*E*_Int_^zpe^), interaction enthalpy (*H*_Int_), and the change in entropy due to dimerization (*S*_Int_) for the relevant dimers in Table S7 in the Supporting Information.

**Figure 7:**
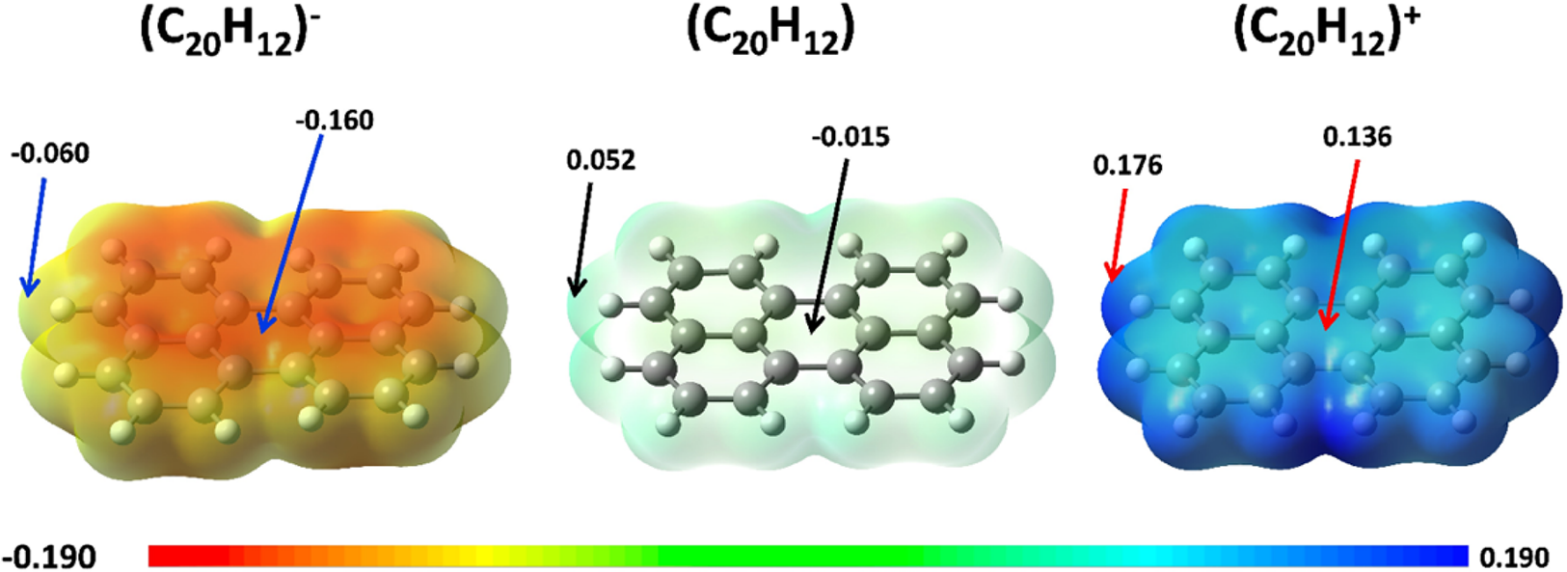
Electrostatic potential, ESP, values at selected points of charged and neutral monomers of perylene. ESP is mapped on an isosurface of the electron density at 0.004 a.u.

The π-electron cloud above and below the plane of a PAH, like perylene, shown in Figure 7, provides an electrostatic potential (ESP) that influences the intermolecular interactions. Charged perylenes therefore experience an asymmetry with respect to the sign of the charge transferred, the ESP being somewhat larger (more repulsive) for negatively charged vs positively charged monomers. This qualitative observation is based on the monomer and cannot be directly used to evaluate the interaction energy differences, but it serves as a qualitative guide for the interpretation of this effect.

**Figure 8:**
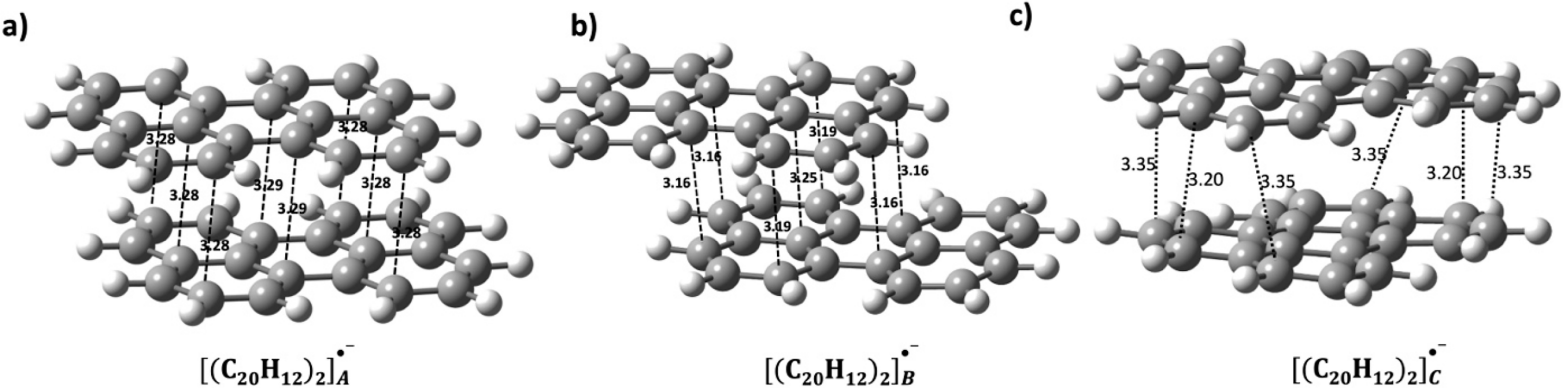
Optimized structures of the monoanionic perylene dimers, (a) [(C_20_H_12_)_2_]_A_^•–^, (b) [(C_20_H_12_)_2_]_B_^•–^, and (c) [(C_20_H_12_)_2_]_C_^•–^. Short contacts are in Å.

According to Table 2, [(C_20_H_12_)_2_]_A_^•–^ ranks as the second most stable dimer for *q* = −1. The optimized structure of [(C_20_H_12_)_2_]_A_^•–^ contains eight short contacts, indicating a more pronounced overlap compared to the seven short contacts observed in the [(C_20_H_12_)_2_]_B_^•–^ (see Figure 8). However, both overlaps maintain θ ≈ 0°, similar to their monocationic counterparts. In the case of [(C_20_H_12_)_2_]_C_^•–^, the optimized structure exhibits θ value of 33.4°, by ∼8° smaller than the monocationic form. The interaction energies between the perylenes in −1 charged dimers are calculated as −12.5, −13.9, and −9.1 kcal/mol for the A-, B-, and C-type dimers, respectively.

**Figure 9:**
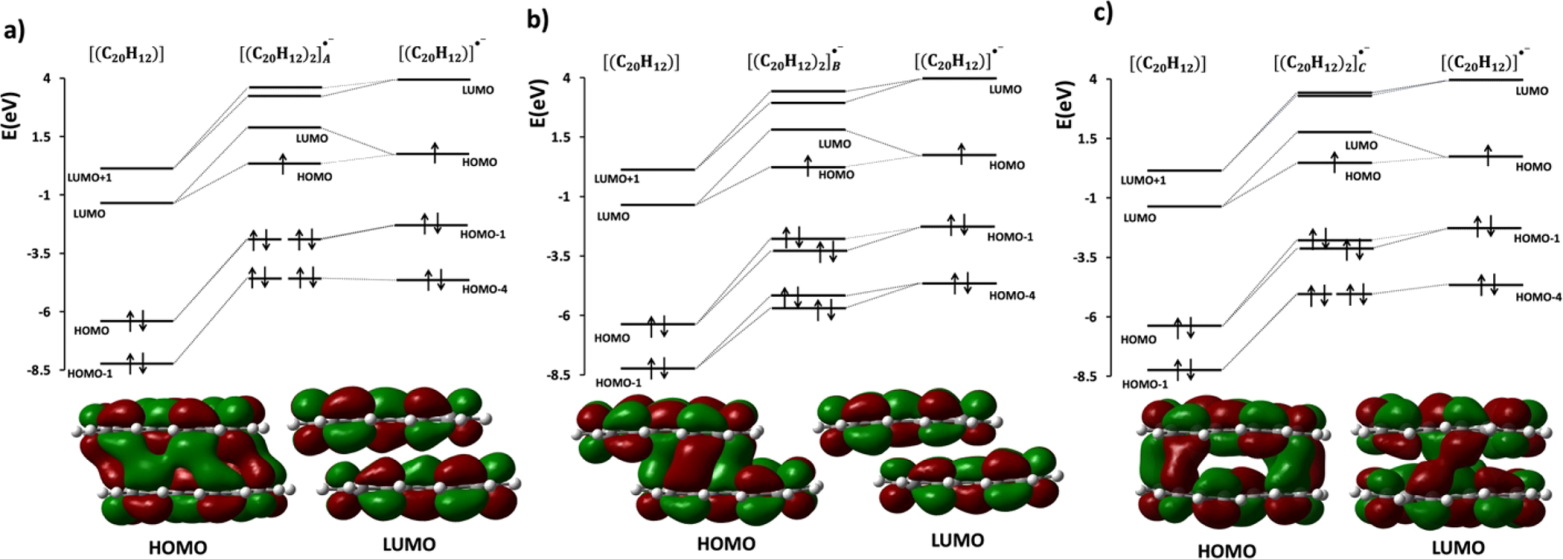
Molecular orbital (MO) diagram for *q* = −1 charged PER dimers. The HOMO of the dimer is a bonding orbital with distinct pancake interaction and contains only one electron. (a) MO interaction diagram of [(C_20_H_12_)_2_]_A_^•–^. (b) MO interaction diagram of [(C_20_H_12_)_2_]_B_^•–^. (c) MO interaction diagram of [(C_20_H_12_)_2_]_C_^•–^.

The HOMO plays a crucial role in monoanionic (*q* = −1) dimers, as expected from Scheme 1. It shows bonding intermolecular orbital overlap, creating a PCB interaction, as seen in Figure 9. The LUMO is antibonding occupied by one electron, resulting in a PBO = 1/2. Consequently, although both monocationic and monoanionic dimers share a PBO value of 1/2, the nature of PCB is markedly different. Based on this analysis, *q* = −1 dimeric perylenes might be possible, *q* = −2 ones are very unlikely to be made.

### 3.6 Perylene Dimers with Overall Charge of *q* = +1: Experiments

The application of the computational modeling is severely hampered in several cases by the complex distribution of the positive charges in cation salts, which represent the majority (24 out of 33) of experimentally determined crystal structures of perylene salts. Some cases are straightforward, such as DIQDEO with the composition of C_20_H_12_^+^, C_20_H_12_, C_32_H_12_BF_24_^–^ with one +1 charge per two perylenes that are crystallographically equivalent. This corresponds to *Q* = +1/2 charge per perylene and *q* = 2*Q* = +1 charge per dimer. At the other end of the complexity of charge distribution is ECINAF (2(C_20_H_12_^+^), 3(C_20_H_12_), Mo_6_O_19_^2–^). This structure contains four different kinds of PCBs. For this reason, a detailed analysis of the charge distribution is necessary. This analysis is possible due to the strong correlation between the experimentally determined BLA. First, we obtained the BLA values for each symmetrically unrelated perylene, as listed in Table 3. Four further systems with large disorder are listed in Table S5. Note that the BLA values are subject to errors as they are based on XRD data. The second step was to use the correlation in Figure 2a to estimate the charges on each perylene. These charge values were then adjusted such that the total charges on all perylenes in one unit cell fully canceled the charges of the counterions, and these charges are given in Table 3.

**Table 3:** BLA Values and Assigned Perylene (PER) Charges, *Q*, in 18 Crystal Structures from the CSD^a^

ref code *Z* and *Z*′	CSD formula, formal charge assignments	BLA [Å]	sym	num	proposed *Q*/*e*	nature of pancake bonds (PCBs)	types of PCB as per Figure 1
XIWQON^20^*Z* = 2, *Z*′ = 0.5	C_20_H_12_^+^, C_20_H_12_, SbCl_6_^–^	–0.373	N	2	+1/2	stacked column, alternating PCB	A
EBUKIW^42^*Z* = 2, *Z*′= 1	3(C_20_H_12_^+^), 3(C_20_H_12_), Mo_12_O_40_P^3–^, CH_2_Cl_2_	–0.636	Y	1	0	alternating column, PCB in tetramer with S–L alternation.	A, A′
		–0.412	N	2	+1/2		
		–0.333	N	2	+1/2		
		–0.364	Y	1	0		
		–0.322	N	2	+1/2		
		–0.317	N	2	+1/2		
		–0.224	N	2	+1		
WERVAR^36^*Z* = 1 , *Z*′ = 1/2	2(C_20_H_12_^+^), C_20_H_12_, 2(C_8_AuF_6_N_2_S_4_^–^)	–0.219	Y	1	+2/3	isolated PER_3_^+2^ trimer with equidistant PCB	A
		–0.239	N	2	+2/3		
RUVGIY^37^*Z* = 2 , *Z*′ = 1	C_20_H_12_^+^, C_20_H_12_, C_16_H_8_CuN_4_S_4_^–^	–0.144	N	2	+1	dimer of PER^+^, one end connected with vdW to PER^0^	A
		–0.654	N	2	0		
ECINEJ^25^*Z* = 1, *Z*′ = 1/2	3(C_20_H_12_^+^), 2(C_20_H_12_), O_19_VW_5_^3–^	0.011	N	2	+1	PER^0^ is isolated, the others form a stacked column of PER_4_^3+^.	F(S), C(L), A(L), C(L)
		–0.236	N	2	+1/2		
		–0.756	Y	1	0		
DIQDEO^21^*Z* = 4 , *Z*′ = 1/2	C_20_H_12_^+^, C_20_H_12_, C_32_H_12_BF_24_^–^	–0.325	N	2	+1/2	stacked column with alternating PCBs and vdW dimers.	B
SEDLIW^43^*Z* = 2, *Z*′ = 0.5	C_20_H_12_^+^, C_20_H_12_, C_8_N_4_PdS_4_^–^	–0.351	N	2	+1/2	equidistant stacked column with PCB	B
SEDLES01^39^*Z* = 2, *Z*′ = 0.5	C_20_H_12_^+^, C_20_H_12_ C_8_AuN_4_S_4_^–^	–0.125	N	2	+1/2	equidistant stacked column with PCB	B
PAJWUS^44^*Z* = 4, *Z*′ = 1	C_20_H_12_^+^, C_20_H_12_, C_8_FeN_4_S_4_^–^	–0.301	N	2	+1/2	equidistant stacked column with PCB	B
ECINAF^25^*Z* = 1 , *Z*′ = 1/2	2(C_20_H_12_^+^), 3(C_20_H_12_), Mo_6_O_19_^2–^	–0.806	Y	1	0	stacked column of (PER_4_)^+2^ with three distinct types of PCB	C, F, C, A
		–0.211	N	2	+1/4		
		–0.486	N	2	+3/4		
PAJXAZ01^38^*Z* = 2, *Z*′ = 1/2	C_20_H_12_^+^, C_20_H_12_, C_8_CoN_4_S_4_^–^	–0.355	N	2	+1/2	stacked equidistant column, PCB	B
ZIBNED^45^*Z* = 2 , *Z*′ = 1	C_20_H_12_^+^, 2(C_20_H_12_), Cl_4_Fe^–^	–0.734	Y	1	0	stacked column of (PER_4_)^+2^ with three distinct types of PCB	A, A′, A″, A
		–0.518	Y	1	0		
		–0.423	N	2	+1/2		
		–0.260	N	2	+1/2		
WIKZOF^40^*Z* = 1 , *Z* = 1/2	C_20_H_12_^+^, 5(C_20_H_12_), F_6_P^–^	–0.635	N	2	0	isolated (PER_2_)^+^ dimer with PCB stacked col., alternating PCB	A A, vdW
		–0.424	N	2	+1/2		
		–0.693	N	2	0		
RIMBOH^39^*Z* = 2 , *Z*′ = 0.5	C_20_H_12_^+^, C_20_H_12_, C_8_N_4_PtS_4_^–^	–0.406	N	24	+1/2	equidistant stacked column with PCB	B
DIWDUL^39^*Z* = 1, *Z*′ = 0.5	C_20_H_12_^+^, C_20_H_12_, C_8_N_4_PtS_4_^–^	–0.3	Y	2	+1/2	equidistance stacked column with PCB	B
EBUKOC^42^*Z* = 2, *Z*′ = 1	3(C_20_H_12_^+^), 3(C_20_H_12_), Mo_12_O_40_P^3–^	–0.311	N	2	+2/3	PER columns separated by perpendicular PER^0^, two distinct (PER^+1/2^)_3_ columns with different PCB packing modes	A, A′, A″, A‴, F
		–0.427	N	2	+2/3		
		–0.503	Y	1	0		
		–0.679	Y	2	0		
		–0.292	N	2	+2/3		
		–0.385	N	2	+2/3		
		–0.453	Y	1	+2/3		
HAKJEI^46^*Z* = 2, *Z*′ = 0.5	[(C_20_H_12_)_3_]^+^, (C_20_H_12_), C_24_Co_3_N_12_S_12_^–^	–0.458	N	2	+1/3	PER_3_, with equidistant PCB, PER^0^ separated neighboring trimers	A
		–0.390	Y	1	+1/3		
		–0.746	Y	1	0		
WERVEV^36^*Z* = 1, *Z*′ = 0.5	2(C_20_H_12_^+^), C_20_H_12_, 2(C_8_F_6_N_2_NiS_4_^–^)	–0.407	Y	1	+2/3	isolated PER trimer with equidistance PCB	A
		–0.426	N	2	+2/3		

a Notes: The crystal structure of DATSUM^47^ (C_20_H_12_^+^, C_8_PtN_4_S_4_^–^) contains isolated PER_2_ dimers sandwiched between C_8_N_4_PtS_4_^–^ anions. The anion–cation interaction indicates strong coordination, and therefore no clear PER charge can be assigned. The crystal structure of EBUKOC contains the heteropolyanion of Mo_12_O_40_P^3–^ (3- charge assigned by Coronado et al.),^42^ but the PER charge assignment is ambiguous. PCB: π–stacking pancake bond. Sym: Is this PER centered on an inversion center? Num: How many PERs of this kind are in the chemical repeat unit? vdW: a vdW gap. S: shorter PCB, L: longer PCB.

**Figure 10:**
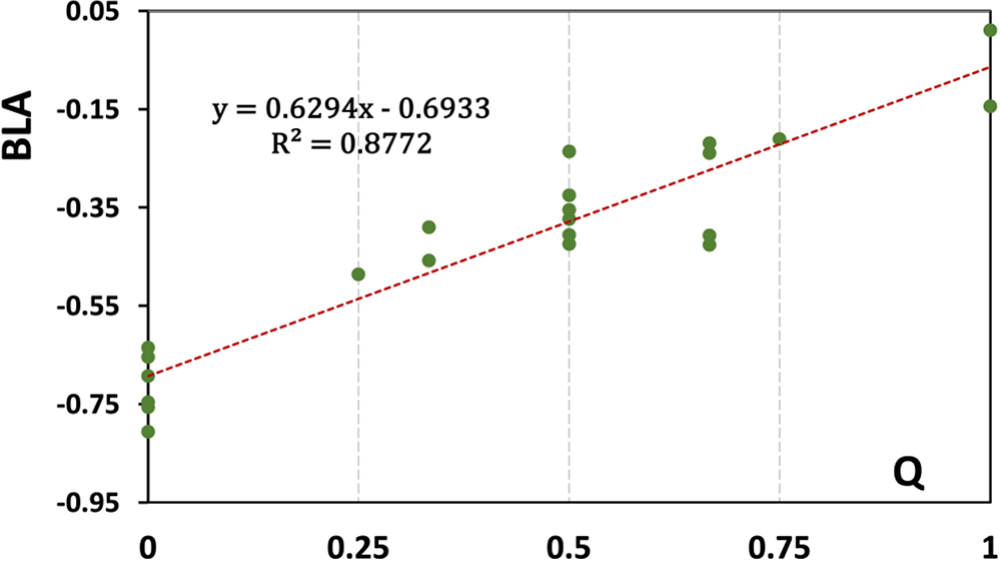
Correlation between assigned perylene charges (*Q*) and experimentally determined BLA as listed in Table 3. Only structures with an *R*-factor of less than 6% are included in this graph. Their CSD ref codes are ECINEJ, DIQDEO, ECINAF, PAJXAZ01, WIKZOF, XIWQON, RIMBOH, WERVAR, RUVGIY, HAKJEI, and WERVEV.

The validity of the obtained charge assignments is confirmed by a correlation between the BLA values and the assigned charges, as shown in Figure 10. This correlation is very satisfactory and forms the basis of the analysis of PCB of perylene salts. Note that in several cases, the charge assigned in this process is *Q* = 0 and the perylenes with such assigned charges do not form pancake bonds with their neighbors. An identical charge assignment except for ECINAF is shown in Figure S13 resulting in a reduction of *R*^2^ in the linear fit to 0.839. The charge assignment of 1/4 and 3/4 in ECINAF in Table 3 implies a charge density wave (CDW),^48^ which corresponds to the fit in Figure 10. This has been replaced by a charge assignment of 1/2 and 1/2 and presented in the correlation in Figure S13. The difference between the two correlations is not sufficiently large to allow for the final determination of the charge distribution although the CDW version is slightly favored.

There is a rich landscape of experimental first neighbor π-stacking data from salts of perylene. Most of these are A-type or B-type. Also, the majority displays *Q* values of +1/2 corresponding to *q* = +1, neatly described by Scheme 1 showing that in this case PCB is driven by HOMO–1 of the interacting dimer. However, a few of the *Q* values deviate from 1/2 ranging from 1/4 to 1. In all these cases, the driving force remains the same HOMO–1 orbital. The differences arise from the different occupancies of the antibonding HOMO of the dimer ranging from 0 (*Q* = 1) to 1.5 (*Q* = 1/4). A small number of structures show F-type overlap, which corresponds to a saddle point on the dimer PES. However, these F-type structures can apparently be stabilized in the crystal structure being structurally close to the D-type. The small number of C-type overlaps found in the crystals correspond to the nearby minimum on the dimer PES.

**Figure 11:**
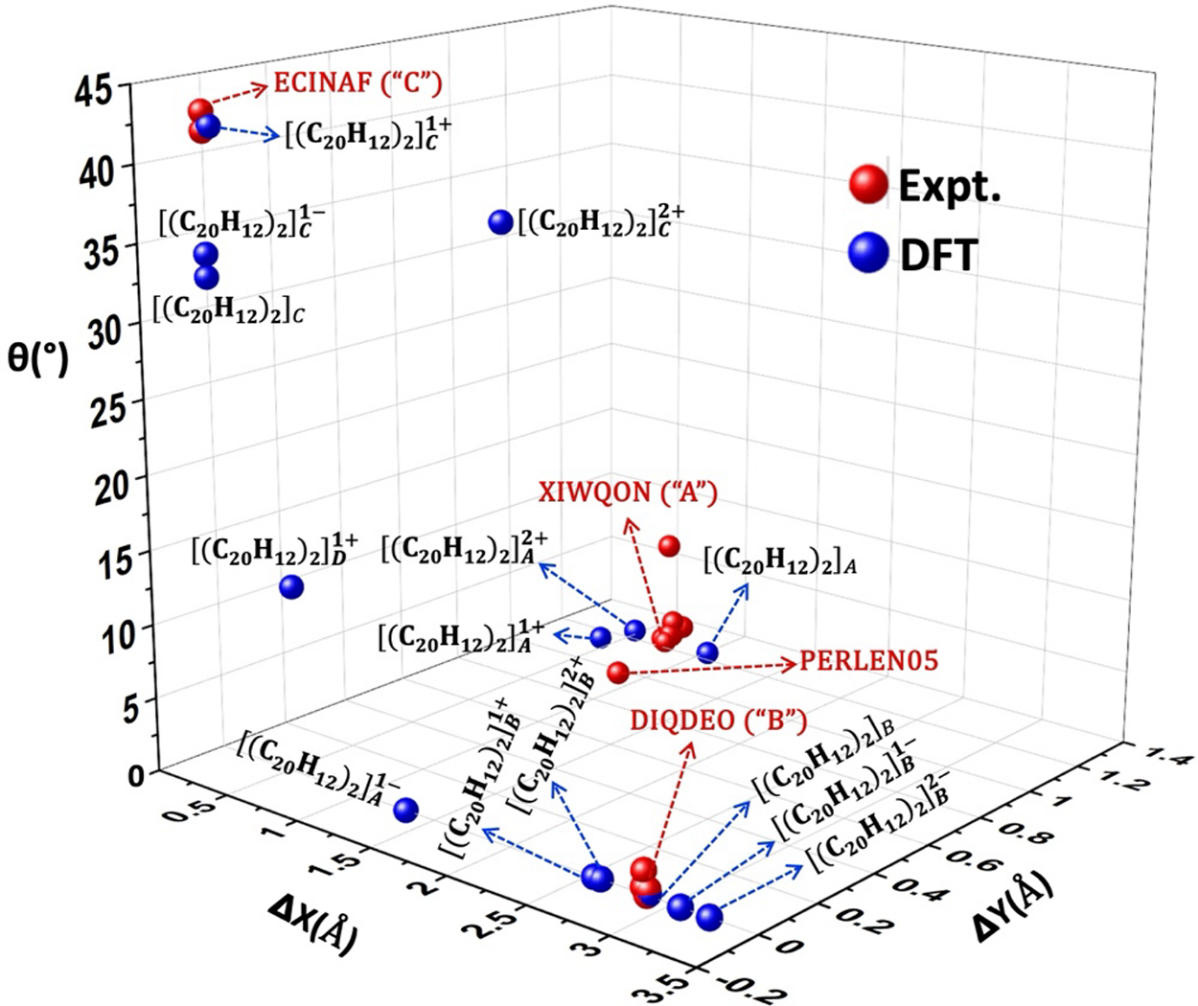
Three-dimensional graph (θ, Δ*X*, and Δ*Y*) of perylene dimer structures. Computed values are in blue, each identified with their charges and structural types according to Table 2. Representative experimental crystal data are in red, each identified with a respective CSD ref code. The crystal of neutral perylene (ref code: PERLEN05)^41^ contains vdW dimers.

An overview of the correlation between computational modeling of perylene PCB as represented by the minima of dimers and a select number of experimentally observed dimer structures are displayed in Figure 11, indicating a satisfactory agreement of first neighbor packing between the experimental and optimized dimers.

For the A-type, the DFT-optimized dimer structures (*q* = +1) closely match the experimental A-type data from XIWQON, (C_20_H_12_^+^, C_20_H_12_, SbCl_6_^–^). Similarly, the DFT-optimized B-type dimers are situated close to the B-type representative crystal structure, DIQDEO (C_20_H_12_^+^, C_20_H_12_, C_32_H_12_BF_24_^–^). As for the C-type dimers, the DFT-optimized *q* = +1 charged dimer structure is very close to the two almost identical experimental points from the structure of ECINAF, (2(C_20_H_12_^+^), 3(C_20_H_12_), Mo_6_O_19_^2–^). There are variations of θ for the differently charged C-type dimers, leading to a distribution for this type compared to the more closely clustered A and B-type dimers.

There are no crystal structures corresponding to the computed structures to compare with, except for *q* = +1. There is also a fourth, the D-type optimized structure, [(C_20_H_12_)_2_]_D_^•+^, occupying a distinct position in Figure 11 with no other nearby experimental points. Since this minimum is very shallow, it is not surprising that there are no perylene π-stacking motifs like the D-type in the CSD.

### 3.7 Spectroscopic Analysis

Further characterization of perylene salts is offered by UV–vis spectral computations for one perylene dimer, namely, [(C_20_H_12_)_2_]_A_^*q*^, at three different *q* values of +1, 0, and −1 following Small et al., who identified a long-wavelength absorption peak accompanying PCB in PLYs.^7^ The experimental UV–vis spectrum of [(C_20_H_12_)_2_]^•+^(SbCl_6_)^−^, which contains A-type [(C_20_H_12_)_2_]_*A*_^•+^ dimers and exhibits a long-wavelength broad shoulder at 544 nm in addition to the main peak around 438 nm.^20^ The DFT-computed spectrum, shown by the black line in Figure S14, showed a good agreement with the experimental results. Previous findings further indicated that the right shoulder was absent in both the neutral and monocationic perylene monomers.^20^ Expanding on this, we computed the UV–vis spectra of both neutral [(C_20_H_12_)_2_]_A_ and anionic [(C_20_H_12_)_2_]_A_^•–^ perylene dimers. The UV–vis spectrum for the neutral dimer in Figure S14 display only one peak around 360 nm, closely resembling the UV–vis spectrum of the neutral perylene monomer. This outcome confirms that the π-stacking geometry alone does not significantly alter the optical properties of perylene, as anticipated due to the absence of significant orbital interactions. However, the absorbance spectrum of the anionic dimer shows distinct differences compared to its neutral counterpart. A right shoulder peak emerged around 560 nm (black line in Figure S14). This peak indicates intermolecular orbital interactions in the anionic dimer, like those observed in the context of PCB in [(C_20_H_12_)_2_]_*A*_^•+^. Experimental verification has to wait until the synthesis of salts with perylenes as anions in pancake bonded structures shown in Scheme 1.

## 4 Conclusions

Perylene, a prototypical closed-shell PAH, does not display pancake bonding in its crystals, only vdW packing. However, its salts display a richness of pancake bonding that should be instructive in the study of the aggregation mechanisms of PAHs in general. So far, the structures of 24 cationic perylene salts have been described in the literature and deposited in the CSD. Perylene as an anion appears in nine crystal structures in the CSD, showing only isolated perylene anions without pancake bonding. The presented computations provide convincing arguments why aggregation should be rare for anionic perylenes, and by extension, probably for other anionic PAHs as well.

On the other hand, cationic perylenes display a wide range of aggregated structures, many showing a variety of pancake bonding, in some cases involving more than one type. Some of these are highly conductive materials, necessitating a better understanding of the structural principles underlying their designs.

We have identified five types of perylene-to-perylene PCB based on the computed PESs of charged perylene dimers, (PER_2_)^*q*^, where *q* is the total charge on the dimer, and *Q* = *q*/2 is the charge per monomer. The work was aided by being able to identify *Q* values in complex crystals based on the excellent correlation found between *Q* and a BLA parameter calculable for each individual perylene from their XRD structures.

The stabilizing effect of PCB occurs due to some radical character of the cationic perylenes, but partial charges suffice to establish such bonding. In fact, *q* = +1 is much more effective than *q* = +2 even though the formal intermolecular PBO is 1/2 in the former, compared to 1 in the latter. Moreover, for negative *q* values, the stabilization provided by PCB is less effective than for positive values, due to the ubiquitous slight negative charge distribution around neutral PAHs.^7,19^ This explains the lack of PCB in the anionic perylene salts.

The qualitative analysis provided in this overview was based on the PES of dimers, not full crystal structures. Why should dimers effectively represent pancake bonding intermolecular interactions of about −8 kcal/mol correctly, when the Coulomb interactions in these salt crystals are larger? The answer lies in the very specific electron sharing nature of pancake bonding. These interactions are very sensitive to small changes of the relative motions of the interacting moieties, while electrostatic interactions, as well as dispersion, are both less specific in terms of a preferred configuration. Once, however, the charge per monomer is high, electrostatics takes over, and pancake bonding becomes less likely. In fact, in the perylene salts, no example has been found with *q* = +2 or −2, even though local minima exist for the dimers in which the relevant SOMO π–orbitals overlap efficiently.

Overall, this study shows how theory explains and drives design of new organic materials by building on the richness of pancake bonding in a prototypical PAH. It is likely that further pancake-bonded ionic dimers and other stacking aggregates including infinite stacks will be made to advance our understanding of the intricate interplay between the relative orientation of PAHs in these salts. It is likely that most of them will be in the *q* = +1 category with *Q* = +1/2 charge on each PAH.

## Supporting Information Available

DFT method selection, optimized structures, free energy profile, spin densities, molecular orbital diagrams, orbital visualization, 3D reaction path, UV–vis spectrum, periodic boundary computations, HOMA (Harmonic Oscillator Model of Aromaticity) data, and optimized coordinates. The Supporting Information is available free of charge at https://pubs.acs.org/doi/10.1021/jacs.3c14065.

DFT method selection, optimized structures, free energy profile, spin densities, molecular orbital diagrams, orbital visualization, 3D reaction path, UV–vis spectrum, periodic boundary computations, HOMA (Harmonic Oscillator Model of Aromaticity) data, and optimized coordinates (PDF)
Movies of structural interconversions (ZIP)
Optimized structures (XYZ)

## Supplementary Material

ja3c14065_si_002.zip

ja3c14065_si_003.xyz
