# Supplementary material for: Structure and Bonding in π-Stacked Perylenes: The Impact of Charge on Pancake Bonding
Source: J Am Chem Soc. 2024 Apr 5;146(15):10465–77. doi: 10.1021/jacs.3c14065 (PMC11027137; doi:10.1021/jacs.3c14065)
Supplement: Supplementary file 1 — ja3c14065_si_001.pdf [file ja3c14065_si_001.pdf]

Supporting Information For

Structure and Bonding in π-Stacked Perylenes: The Impact of Charge on Pancake Bonding

Rameswar Bhattacharjee[1]*, Henry Jervis[1], Megan E. McCormack,[2] Marina A. Petrukhina[2], Miklos Kertesz[1]*
[1]Chemistry Department and Institute of Soft Matter, Georgetown University
37th and O Streets, NW, Washington DC 20057-1227, USA
[2]Department of Chemistry, University at Albany, State University of New York, 1400 Washington Avenue, Albany, NY 12222, USA
*E-mail: rb1820@georgetown.edu , kertesz@georgetown.edu

Table of Contents

1. Selection of the DFT method. (Tables S1-S3)
2. Structural parameters of 19 pancake bonded perylene salts. (Table S4)
3. Tables of BLA values and charge assignments. (Table S5)
4. BLA values and assigned charges for nine anionic perylene crystals. (Table S6)
5. DelG values. (Table S7)
6. Optimized structures of $[(C_{20}H_{12})_2]_A^{\bullet+}$ and $[(C_{20}H_{12})_2]_B^{\bullet+}$. (Figures S1-S2).
7. Changes of HOMO-1 orbital during transformation from $[(C_{20}H_{12})_2]_A^{\bullet+}$ (A) to $[(C_{20}H_{12})_2]_B^{\bullet+}$ (B). (Figure S3)
8. Molecular orbital diagram and orbitals of $[(C_{20}H_{12})_2]_D^{\bullet+}$. (Figure S4)
9. 3D reaction path. (Figure S5)
10. Spin density of transition states. (Figure S6)
11. **Δ**X and **Δ**Y translational values of perylene dimers. (Figure S7)
12. Optimized structures of mono-anionic perylene dimers. (Figure S8-S10)
13. Spin densities of mono-anionic dimers. (Figure S11)
14. Optimized structures of neutral dimers. (Figure S12)
15. Alternative charge assignment for. (Figure S13)
16. Charge effects on the number of unpaired electrons.
17. UV-vis spectrum prediction. (Figure S14)
18. Periodic boundary computations. (Figures S15-17)
19. Assessment of aromaticity by HOMA. (Table S8)
20. QTAIM analysis. (Tables S9-11)
21. Local vibrational mode force constant analysis. (Table S8)
22. Optimized Coordinates are provided in a separate XYZ file.

Selection of the DFT method

There is a wide selection of high-level quantum chemical methods and DFTs available for describing pancake bonded aggregates. In this work we opted for M05-2X/6-311G(d), a method that includes some dispersion effects.[1] This choice is based on a previous in-depth analysis of four pancake-bonded π-dimers.[2] The results of this study consistently demonstrated that the M05-2X outperformed other modern DFT functional when compared to the highly accurate multi reference average quadratic coupled cluster MR-AQCC method.[3] This evaluation of more than 50 contemporary DFT functionals, including those incorporating dispersion correction, for the pancake-bonded dimers consistently confirmed that M05-2X exhibited the lowest error in terms of optimized geometry, interaction energy, and a few other parameters.

In response to a reviewer's suggestion, we are presenting the impact of including D3 dispersion terms[4] on our results, particularly on binding energies and optimized geometries. Upon comparing the interaction energies for different types of charged dimers, we observed that the additional D3 correction led to larger (more negative) interaction energies by approximately 6.0 kcal/mol for most dimers as listed below. The relatively large interaction energy for the uncharged (vdW) dimers at ~20 kcal/mol is exaggerated further justifying the theory without the D3 terms in this application.

Table S1: Computed interaction energies for the various perylene dimers at various charges employing M05-2X/6-311G(d) and M05-2X-D3/6-311G(d).

Optimized Dimer	EInt (M05-2X, kcal/mol)	EInt (M05-2X-D3, kcal/mol)
$[\boldsymbol{C_{20}H_{12}}]_A^{+2}$	+18.7	+12.7
$[\boldsymbol{C_{20}H_{12}}]_B^{+2}$	+17.3	+11.6
$[\boldsymbol{C_{20}H_{12}}]_C^{+2}$	+18.8	+12.7
$[\boldsymbol{C_{20}H_{12}}]_A^{+1}$	-19.6	-25.7
$[\boldsymbol{C_{20}H_{12}}]_B^{+1}$	-20.4	-26.1
$[\boldsymbol{C_{20}H_{12}}]_C^{+1}$	-19.9	-26.2
$[\boldsymbol{C_{20}H_{12}}]_A$	-13.5	-19.6
$[\boldsymbol{C_{20}H_{12}}]_B$	-11.1	-16.8
$[\boldsymbol{C_{20}H_{12}}]_C$	-13.6	-19.8
$[\boldsymbol{C_{20}H_{12}}]_A^{-1}$	-12.5	-18.5
$[\boldsymbol{C_{20}H_{12}}]_B^{-1}$	-13.9	-19.3
$[\boldsymbol{C_{20}H_{12}}]_C^{-1}$	-9.1	-15.3
$[\boldsymbol{C_{20}H_{12}}]_B^{-2}$	+26.4	+20.9

Concerning the optimized geometries, the differences between the two methods (without and with the D3 terms) are small and are slightly longer with the D3 terms included, in agreement with Ref. [1]. These data are summarized in the two tables below. None of the conclusions are affected by the inclusion of the D3 term.

Table S2: Optimized intermolecular carbon-carbon (CC) short contacts in angstroms (Å) for A-type perylene dimers obtained using M05-2X/6-311G(d) and M05-2X-D3/6-311G(d).

**CC short contacts in Å**	$[C_{20}H_{12}]_A^{+1}$ **M05-2X**	$[C_{20}H_{12}]_A^{+1}$ **M05-2X-D3**	$[C_{20}H_{12}]_A$ **M05-2X**	$[C_{20}H_{12}]_A$ **M05-2X-D3**	$[C_{20}H_{12}]_A^{-1}$ **M05-2X**	$[C_{20}H_{12}]_A^{-1}$ **M05-2X-D3**
$(C\cdots C)_1$	3.362	3.365	3.360	3.357	3.282	3.286
$(C\cdots C)_2$	3.349	3.355	3.328	3.326	3.280	3.287
$(C\cdots C)_3$	3.299	3.306	3.326	3.327	3.278	3.285
$(C\cdots C)_4$	3.272	3.278	3.288	3.290	3.288	3.295
$(C\cdots C)_5$	3.271	3.277	3.289	3.289	3.288	3.295
$(C\cdots C)_6$	3.299	3.306	3.326	3.327	3.280	3.286
$(C\cdots C)_7$	3.350	3.355	3.327	3.327	3.280	3.287
$(C\cdots C)_8$	3.362	3.365	3.359	3.357	3.280	3.285

Table S3: Optimized intermolecular carbon-carbon (CC) short contacts in angstroms (Å) for A-type perylene dimers obtained using M05-2X/6-311G(d) and M05-2X-D3/6-311G(d).

**CC short contacts in Å**	$[C_{20}H_{12}]_B^{+1}$ **M05-2X**	$[C_{20}H_{12}]_B^{+1}$ **M05-2X-D3**	$[C_{20}H_{12}]_B$ **M05-2X**	$[C_{20}H_{12}]_B$ **M05-2X-D3**	$[C_{20}H_{12}]_B^{-1}$ **M05-2X**	$[C_{20}H_{12}]_B^{-1}$ **M05-2X-D3**
$(C\cdots C)_1$	3.217	3.229	3.312	3.320	3.160	3.161
$(C\cdots C)_2$	3.217	3.228	3.312	3.321	3.159	3.161
$(C\cdots C)_3$	3.276	3.288	3.391	3.397	3.196	3.199
$(C\cdots C)_4$	3.258	2.271	3.377	3.389	3.251	3.255
$(C\cdots C)_5$	3.276	3.285	3.388	3.397	3.194	3.199
$(C\cdots C)_6$	3.217	3.229	3.310	3.320	3.160	3.162
$(C\cdots C)_7$	3.217	3.229	3.313	3.321	3.161	3.162

Table S4. Pancake bonded (PCB) cationic perylenes from the Cambridge Structural Database (CSD) listing all 24 structures.

Refcode (and reference)	Chemical formula	Contains type A, B, C, or F PCBs (number of short C···C contacts)	PCB parameters[a]	Notes
XIWQON[5]	$[(C_{20}H_{12})_2]^{\bullet+}(SbCl_6)^-)$	A (8)	θ= 0.24, **Δ**X= 0.699<br>**Δ**Y= 1.230, **Δ**Z_{Avg}= 3.309<br>**Δ**Z_O= 3.334	R=3.04%
EBUKIW[6]	$3(C_{20}H_{12}^+)$, $3(C_{20}H_{12})$, $Mo_{12}O_{40}P^{3-}$, CH_2Cl_2	A (8)<br>A' (1)	θ= 7.28, **Δ**X= 0.711<br>**Δ**Y= 1.250, **Δ**Z_{Avg}= 3.372<br>**Δ**Z_O= 3.375	R = 7.3%
WERVAR[7]	$2(C_{20}H_{12}^+)$, $C_{20}H_{12}$, $2(C_8AuF_6N_2S_4^-)$	A (2)	θ= 0.53, **Δ**X= 0.723<br>**Δ**Y= 1.246, **Δ**Z_{Avg}= 3.417<br>**Δ**Z_O= 3.449	R= 2.58%
RUVGIY [8]	$C_{20}H_{12}^+$, $C_{20}H_{12}$, $C_{16}H_8CuN_4S_4^-$	A (2)	θ= 0.34, **Δ**X= 0.726<br>**Δ**Y= 1.239, **Δ**Z_{Avg}= 3.436<br>**Δ**Z_O= 3.419	R= 5.49%
CUWBIF[9]	$C_{20}H_{12}^+$, $5(C_{20}H_{12})$, ClO_4^-	A (2)	θ= 0.17, **Δ**X= 0.73,<br>**Δ**Y= 1.224, **Δ**Z_{Avg}= 3.430<br>**Δ**Z_O= 3.460	R =6.3%
ECINEJ[10]	$3(C_{20}H_{12}^+)$, $2(C_{20}H_{12})$, $O_{19}VW_5^{3-}$	F (20)<br>C (1)<br>A (2)	C: θ= 41.31<br>**Δ**X= 0, **Δ**Y= 0<br>**Δ**Z_O= 3.433<br>A: θ= 0.41<br>**Δ**X= 0.727, **Δ**Y= 1.253<br>**Δ**Z_{Avg}= 3.481<br>**Δ**Z_O= 3.404	R = 5.62%
DIQDEO[11]	$C_{20}H_{12}^+$, $C_{20}H_{12}$, $C_{32}H_{12}BF_{24}^-$	B (7)	θ= 1.53, **Δ**X= 2.831<br>**Δ**Y= 0, **Δ**Z_{Avg}= 3.322,<br>**Δ**Z_O= 3.327	R = 5.55%
SEDLIW[12]	$C_{20}H_{12}^+$, $C_{20}H_{12}$, $C_8N_4PdS_4^-$	B (7)	θ= 0.13, **Δ**X= 2.861,<br>**Δ**Y= 0, **Δ**Z_{Avg}= 3.327<br>**Δ**Z_O= 3.355	R= 7.0 %
SEDLES01[13]	$C_{20}H_{12}^+$, $C_{20}H_{12}$ $C_8AuN_4S_4^-$	B (7)	θ= 0.41<br>**Δ**X= 2.853, **Δ**Y= 0<br>**Δ**Z_{Avg}= 3.292<br>**Δ**Z_O= 3.330	R= 6.94 %

EBUKUI[6]	$4(C_{20}H_{12}^{+})$, $5(C_{20}H_{12})$, $2(O_{40}SiW_{12}^{4-})$, $4(C_{16}H_{36}N^{+})$	B (various)	θ= 0.40, **Δ**X= 2.858<br>**Δ**Y= 0, **Δ**Z_{Avg}= 3.245<br>**Δ**Z_{O}= 3.240	R= 14.5%
PAJWUS[14]	$C_{20}H_{12}^{+}$, $C_{20}H_{12}$, $C_8FeN_4S_4^{-}$	B (4)	θ= 0.48<br>**Δ**X= 2.829, **Δ**Y= 0<br>**Δ**Z_{Avg}= 3.391<br>**Δ**Z_{O}= 3.431	R= 11.1%
ECINAF[10]	$2(C_{20}H_{12}^{+})$, $3(C_{20}H_{12})$, $Mo_6O_{19}^{2-}$	C (5)<br>A (2)<br>F (5)	C: θ= 42.56<br>**Δ**X= 0, **Δ**Y= 0<br>**Δ**Z_{O}= 3.430<br>A: θ= 0.10<br>**Δ**X= 0.709, **Δ**Y= 1.238<br>**Δ**Z_{Avg}= 3.506,<br>**Δ**Z_{O}= 3.418	R= 3.5%
PAJXAZ01[15]	$C_{20}H_{12}^{+}$, $C_{20}H_{12}$, $C_8CoN_4S_4^{-}$	B (6)	θ= 0.04<br>**Δ**X= 2.850<br>**Δ**Y= 0, **Δ**Z_{Avg}= 3.353<br>**Δ**Z_{O}= 3.335	R= 4.4%
DATSUM[16]	$C_{20}H_{12}^{+}$, $C_8PtN_4S_4^{-}$	A (2)	θ= 1.31,<br>**Δ**X= 0.718, **Δ**Y= 1.263<br>**Δ**Z_{Avg}= 3.507<br>**Δ**Z_{O}= 3.355	R= 7.8%
ZIBNED[17]	$C_{20}H_{12}^{+}$, $2(C_{20}H_{12})$, Cl_4Fe^{-}	A (8)<br>A’ (1)<br>A”(2)	A: θ= 0.14<br>**Δ**X= 0.729, **Δ**Y= 1.280<br>**Δ**Z_{Avg}= 3.420<br>**Δ**Z_{O}= 3.431<br>A’: θ= 0.88<br>**Δ**X= 0.693, **Δ**Y= 1.294<br>**Δ**Z_{Avg}= 3.414<br>**Δ**Z_{O}= 3.405<br>A’’: θ= 0.75<br>**Δ**X= 0.733, **Δ**Y= 1.295<br>**Δ**Z_{Avg}= 3.373<br>**Δ**Z_{O}= 3.370	R = 6.49%
RIMBOH[13]	$C_{20}H_{12}^{+}$, $C_{20}H_{12}$, $C_8N_4PtS_4^{-}$	B (7)	θ= 0.14, **Δ**X= 2.817<br>**Δ**Y= 0.0, **Δ**Z_{Avg}= 3.30<br>**Δ**Z_{O}= 3.325	R = 1.91%
WIKZOF[18]	$C_{20}H_{12}^{+}$, $C_{20}H_{12}$, F_6P^{-}	A (4)	θ= 1.12, **Δ**X= 0.687<br>**Δ**Y= 1.176<br>**Δ**Z_{Avg}= 3.396<br>**Δ**Z_{O}= 3.407	R = 5.86%
PERYHA[19]	$C_{20}H_{12}^{+}$, $C_{20}H_{12}$, F_6P^{-}, $0.1(F_6P)$, $0.8(CH_2Cl_2)$	B (6)	θ= 0.41, **Δ**X= 2.833<br>**Δ**Y= 0.0	R = 14.8%

			$\boldsymbol{\Delta}Z_{Avg}$= 3.378<br>$\boldsymbol{\Delta}Z_O$= 3.474	
PERYHB[19]	$C_{20}H_{12}^+$, $C_{20}H_{12}$, AsF_6^-, 0.1(AsF_6), 0.7(CH_2Cl_2)	B (2)	θ= 0.6, $\boldsymbol{\Delta}$X= 2.833<br>$\boldsymbol{\Delta}$Y= 0.0<br>$\boldsymbol{\Delta}Z_{Avg}$= 3.404<br>$\boldsymbol{\Delta}Z_O$= 3.428	R = 9.8%
CUWBEB[9]	$C_{20}H_{12}^+$, 2($C_{20}H_{12}$), ClO_4^-	A (1)	θ= 0.31<br>$\boldsymbol{\Delta}$X= 0.714, $\boldsymbol{\Delta}$Y= 1.246,<br>$\boldsymbol{\Delta}Z_{Avg}$= 3.475	R= 11.6%
DIWDUL[13]	$C_{20}H_{12}^+$, $C_{20}H_{12}$, $C_8N_4PtS_4^-$	B (12)	θ= 0.25<br>$\boldsymbol{\Delta}$X= 2.830, $\boldsymbol{\Delta}$Y= 0, $\boldsymbol{\Delta}Z_O$= 3.323	R= 7.75%
EBUKOC[6]	3($C_{20}H_{12}^+$), 3($C_{20}H_{12}$), $Mo_{12}O_{40}P^{3-}$, C_2H_3N	A (8)<br>F (2)<br>A’ (4)<br>A’’ (2)	A: θ= 0.13<br>$\boldsymbol{\Delta}$X= 0.689, $\boldsymbol{\Delta}$Y= 1.195,<br>$\boldsymbol{\Delta}Z_{Avg}$= 3.361<br>F: θ= 4.43<br>$\boldsymbol{\Delta}$X= 0, $\boldsymbol{\Delta}$Y= 0.57,<br>$\boldsymbol{\Delta}Z_{Avg}$= 3.505<br>A’: θ= 0.13<br>$\boldsymbol{\Delta}$X= 0.675, $\boldsymbol{\Delta}$Y= 1.185,<br>$\boldsymbol{\Delta}Z_{Avg}$= 3.407<br>A’’: θ= 0.88<br>$\boldsymbol{\Delta}$X= 0.685, $\boldsymbol{\Delta}$Y= 1.178,<br>$\boldsymbol{\Delta}Z_{Avg}$= 3.425	R= 4.06%
HAKJEI[20]	[$(C_{20}H_{12})_3$]$^+$, ($C_{20}H_{12}$), $C_{24}Co_3N_{12}S_{12}^-$	A (1)	θ= 0.12<br>$\boldsymbol{\Delta}$X= 0.662, $\boldsymbol{\Delta}$Y= 1.153,<br>$\boldsymbol{\Delta}Z_O$= 3.444	R = 4.7%
WERVEV[7]	2($C_{20}H_{12}^+$), $C_{20}H_{12}$, 2($C_8F_6N_2NiS_4^-$)	A (0)	θ= 0.25<br>$\boldsymbol{\Delta}$X= 0.660, $\boldsymbol{\Delta}$Y= 1.183,<br>$\boldsymbol{\Delta}Z_{Avg}$= 3.440	R = 4.6%

[a]PCB parameters (if several, only the shortest are shown) θ, **Δ**X, **Δ**Y, and **Δ**Z.
$\boldsymbol{\Delta}Z_{Avg}$: average of overlapping carbons)
$\boldsymbol{\Delta}Z_O$: an alternative measure of the characteristic interlayer distance was determined based on Scheme 3 in the main text. We used Mercury (part of the CSD program package) to determine a centroid of the center ring of one perylene to act as the origin and measured the distance between that and the neighboring perylene.

Table S5. BLA values and assigned cationic perylene charges from six crystal structures from the Cambridge Structural Database (CSD) with large disorder.

REFCODEZ and Z'	CSD Formula, Some of the charge assignments are formal only	BLA [Å]	Symm	How Many of this PER in U.C.	Proposed Q	Nature of PCBs among perylenes if present	Types of PCB according to Fig. 1.
PERYHA[19] Z=1 Z'=0.13	$C_{20}H_{12}^{+}$, $C_{20}H_{12}$, 0.8(CH_2Cl_2), F_6P^{-}, 0.1(F_6P^{-})	-0.894	Y	4	+1/2	Equidistant stacked column, PCB	B
CUWBIF[9] Z=1, Z'=0.5	$C_{20}H_{12}^{+}$, 5($C_{20}H_{12}$), ClO_4^{-}	-0.401	N	1	+0.5	Isolated $(PER_2)^{+}$ dimer with PCB, disordered	A
		-0.621	N	4	0		
		-0.560	N	1	+0.5		
EBUKUI[6] Z=1 Z'=1/2	4($C_{20}H_{12}^{+}$), 5($C_{20}H_{12}$), 4($C_{16}H_{36}N^{+}$), 2($O_{40}SiW_{12}$)$^{4-}$	-0.278<br>-0.288<br>-0.278<br>-0.300<br>-0.667	N<br>N<br>N<br>N<br>Y	2<br>2<br>2<br>2<br>1	+0.5<br>+0.5<br>+0.5<br>+0.5<br>0	Stacked column of PERs with alternating arrangements of five kinds of PCBs and isolated PER_2	B, C
PERYHB[19] Z=1 Z'=1/8	$C_{20}H_{12}^{+}$, $C_{20}H_{12}$, AsF_6^{-}, 0.1(AsF_6^{-}), 0.7(CH_2Cl_2)	-0.270	Y	3	+1/2	Equidistant stacked column, PCB	B
CUWBEB[9] Z= 2 Z'= 1	$C_{20}H_{12}^{+}$, 2($C_{20}H_{12}$), ClO_4^{-}	-0.562	Y	1	0	BLA data are not very reliable.	A
		-0.199	Y	1	?		
		-0.111	N	1	?		
		0.206	N	1	?		
DATSUM[16] Z=2, Z'=1	$C_{20}H_{12}^{+}$, $C_8N_4PtS_4^{-}$	-0.349	N	1	+1	Isolated $(PER_2)^{+}$ dimer with PCB	A

Table S6. BLA values and assigned charges for nine anionic perylene crystal structures from the Cambridge Structural Database (CSD)

CSD REFCODE, Z, and Z'	CSD Formula	BLA	Symmetry	Molecule Configuration	Q=	R-factor [%]
HEDXIX[21] Z=4, Z'=1	$2(C_{12}H_{28}NaO_6^{+})$, $C_{20}H_{12}^{2-}$	0.463	N	Isolated	-2	5.4
HEDXUJ[21] Z=4, Z'=1/2	$C_{16}H_{36}NaO_8^{+}$, $C_{20}H_{12}^{-}$, $C_{20}H_{12}$	-0.625	Y	Isolated	0	4.47
		-0.104	Y		-1	
HEDXUJ01[22] Z=4, Z'=1/2	$C_{16}H_{36}NaO_8^{+}$, $C_{20}H_{12}^{-}$, $C_{20}H_{12}$	-0.625	Y	Isolated	0	4.03
		-0.104	Y		-1	
HITHAT[22] Z=4, Z'=1/2	$C_{12}H_{30}LiO_6$+, $C_{20}H_{12}^{-}$	-0.079	Y	Isolated	-1	4.39
HITHEX[22] Z=4, Z'=1	$C_{16}H_{36}KO_8^{+}$, $C_{20}H_{12}^{-}$	-0.188	N	Treat it as Isolated	-1	4.28
HITHIB[22] Z=8, Z'=1/2	$C_{20}H_{44}CsO_{10}^{+}$, $C_{20}H_{12}^{-}$	-0.113	Y	Isolated	-1	4.13
SUXNEG[23] Z=4, Z'=1/2	$C_{16}H_{34}NaO_8^{+}$, $C_{20}H_{12}^{-}$	-0.128	Y	Isolated	-1	3.35
SUXPEI[23] Z=8, Z'=2	$C_{20}H_{40}KO_8$, $C_{20}H_{12}$	-0.141	N	Isolated BLA is average	0	6.56
SUXPIM[23] Z=2, Z'=1	$C_{16}H_{34}LiO_8$, $1.5(C_{20}H_{12})$	-0.188	N	Isolated	0	5.65
		-0.619	Y	Isolated	0	

Table S7. Interaction energy (E_{Int}), zero-point energy (ZPE) corrected Interaction energy (E_{Int}^{zpe}), free energy of interaction (G_{Int}), interaction enthalpy (H_{Int}), and entropy change for the dimerization (S_{Int}) of the dimers.

Optimized Dimer	E_{Int} (M052X, kcal/mol)	E_{Int}^{zpe} (M052X, kcal/mol)	G_{Int} (M052X, kcal/mol)	H_{Int} (M052X, kcal/mol)	S_{Int} (M052X, cal mol^{-1} K^{-1})
$[C_{20}H_{12}]_A^{+1}$	-19.6	-19.5	-9.0	-20.1	-37.2
$[C_{20}H_{12}]_B^{+1}$	-20.4	-20.3	-10.1	-20.8	-35.9
$[C_{20}H_{12}]_C^{+1}$	-19.9	-19.8	-9.1	-20.5	-38.4
$[C_{20}H_{12}]_D^{+1}$	-16.9	-17.1	-7.1	-17.6	-35.2
$[C_{20}H_{12}]_A$	-13.5	-13.7	-5.8	-13.1	-24.2
$[C_{20}H_{12}]_B$	-11.1	-11.2	-5.1	-10.5	-17.9
$[C_{20}H_{12}]_C$	-13.6	-13.7	-5.4	-13.2	-26.0
$[C_{20}H_{12}]_A^{-1}$	-12.5	-12.7	-2.8	-12.6	-32.9
$[C_{20}H_{12}]_B^{-1}$	-13.9	-13.5	-3.3	-13.6	-34.9
$[C_{20}H_{12}]_C^{-1}$	-9.1	-9.8	-0.4	-9.7	-31.5

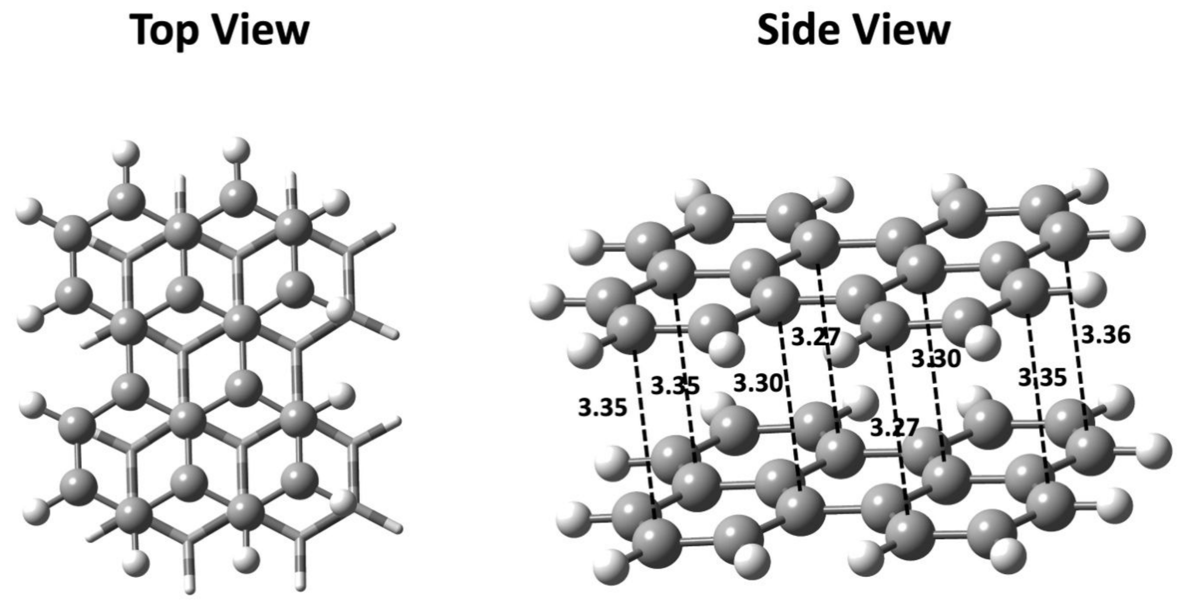

$[(\mathbf{C_{20}H_{12}})_2]_A^{\bullet +}$

Figure S1. Optimized structure (top and side view) of $[(C_{20}H_{12})_2]_A^{\bullet +}$ highlighting eight short contacts (in Å). [5]

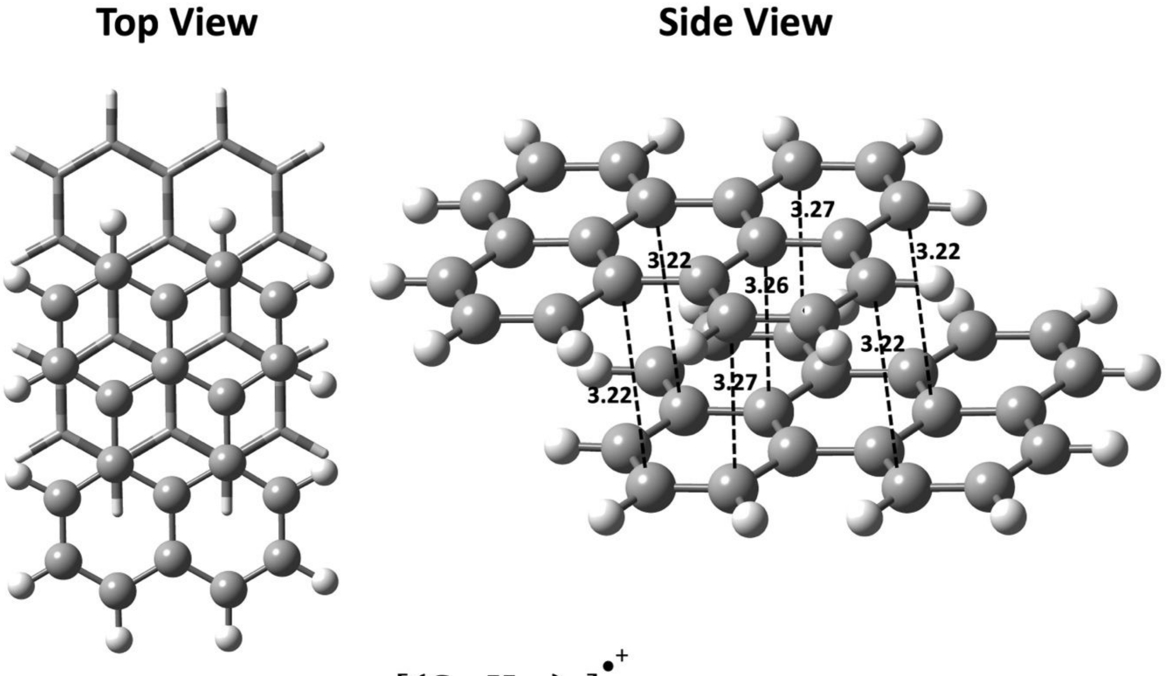

$[(\mathbf{C_{20}H_{12}})_2]_B^{\bullet +}$

Figure S2. Optimized structure (top and side view) of $[(C_{20}H_{12})_2]_B^{\bullet +}$ and highlights seven short-range contacts, (in Å).[5]

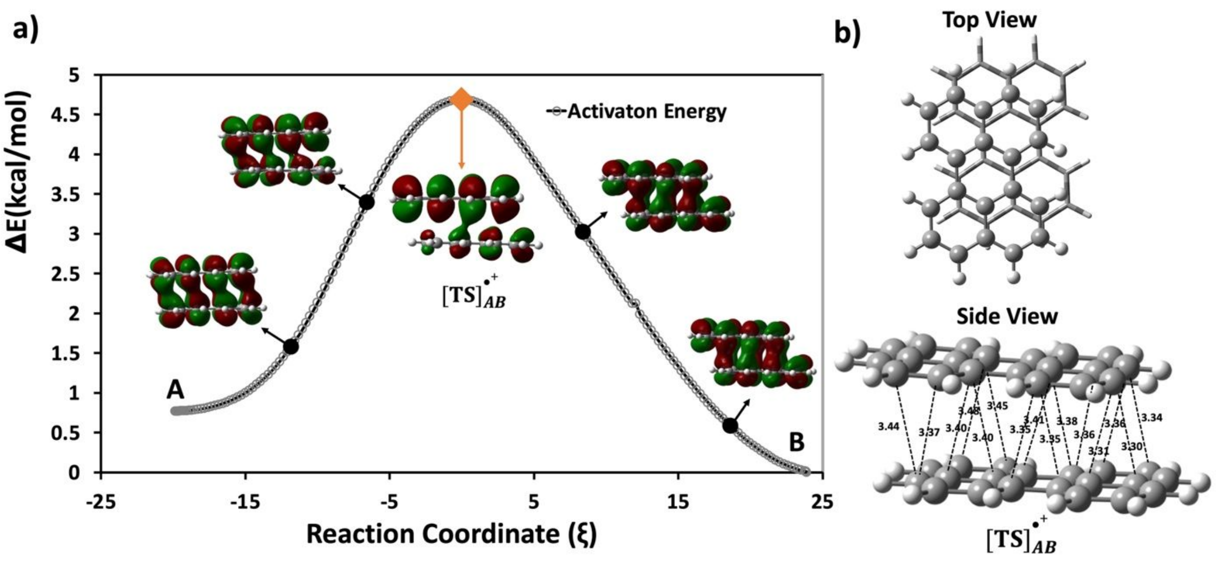

Figure S3. The development of the HOMO-1 orbital during the transition from structure $[(C_{20}H_{12})_2]_A^{\bullet+}$ to structure$[(C_{20}H_{12})_2]_B^{\bullet+}$.[3]

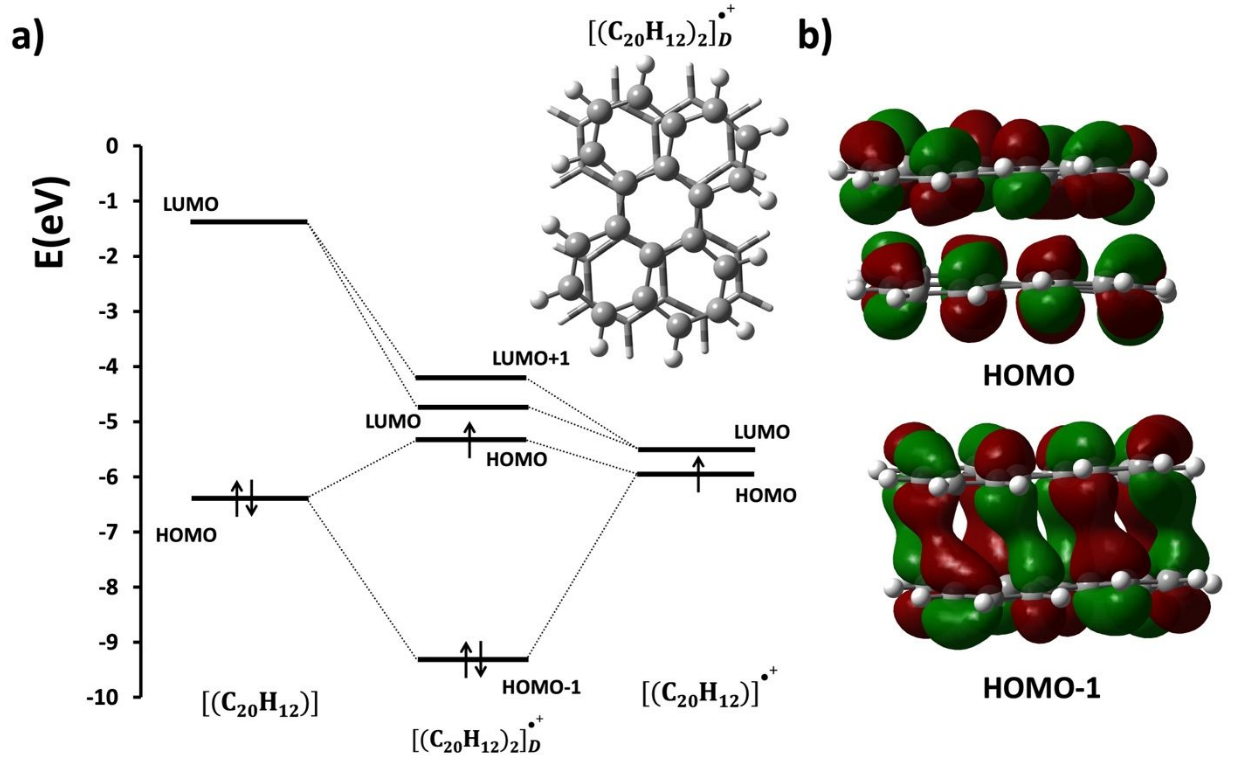

Figure S4. a) Molecular orbital diagram in for $[(C_{20}H_{12})_2]_D^{\bullet+}$, an intermediate characterized in the potential energy surface in the Figures 5 in the main text and Figure S5. b) HOMO-1 and HOMO orbitals for the same.

3D reaction path

The full reaction path is shown Figure S5 incorporates all three parameters ΔX, ΔY, and θ together. The gray line and gray points illustrate the change in the relative orientation of perylene molecules, passing through the unique orange points representing the TSs. Throughout the transition from A to B, the path stays on the XY plane. However, when transitioning from an A-type dimer to a C-type dimer, the process becomes more intricate as θ starts to deviate from zero. This transformation necessitates both the translation and rotation of one perylene molecule over another. The journey begins from $[(C_{20}H_{12})_2]_A^{\bullet+}$ (ΔX=0.31Å, ΔY=1.15Å, θ=0.0°) to $[(C_{20}H_{12})_2]_C^{\bullet+}$ (ΔX=0.0 Å, ΔY=0.0 Å, θ =41.7°) via an intermediate D((ΔX=0.5 Å, ΔY=0.0 Å, θ =12.1°). The most notable changes in θ occur during the transformation from D to C, where the θ value ultimately reaches 41.7° in the resulting C-type dimer. A movie depicting the complete transformation along the reaction path is provided in the supporting information.

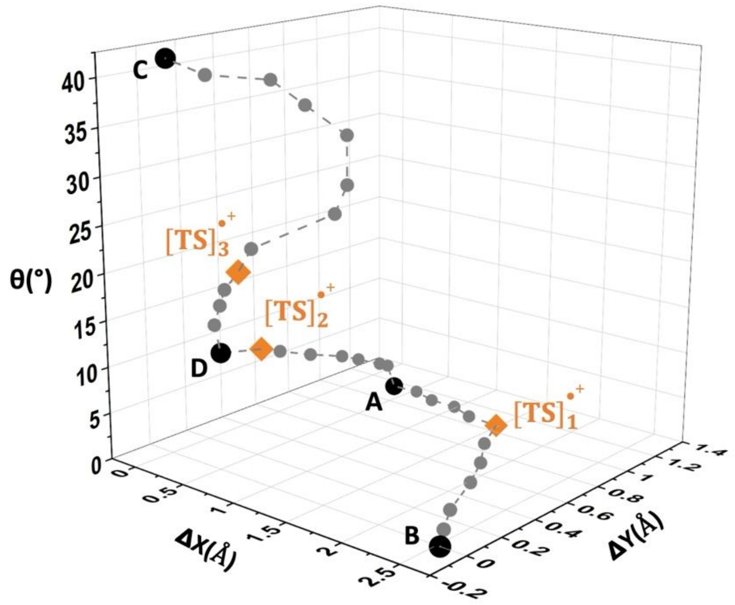

Figure S5. Optimized **Δ**X, **Δ**Y, and θ values of +1 charged perylene dimers. Grey points and the grey connecting line indicate the transformation pathway from the optimized structure of $[(C_{20}H_{12})_2]_A^{\bullet+}$ (A) to that of $[(C_{20}H_{12})_2]_B^{\bullet+}$ (B) and $[(C_{20}H_{12})_2]_C^{\bullet+}$ (C) through the three transition structures and one intermediated (D).

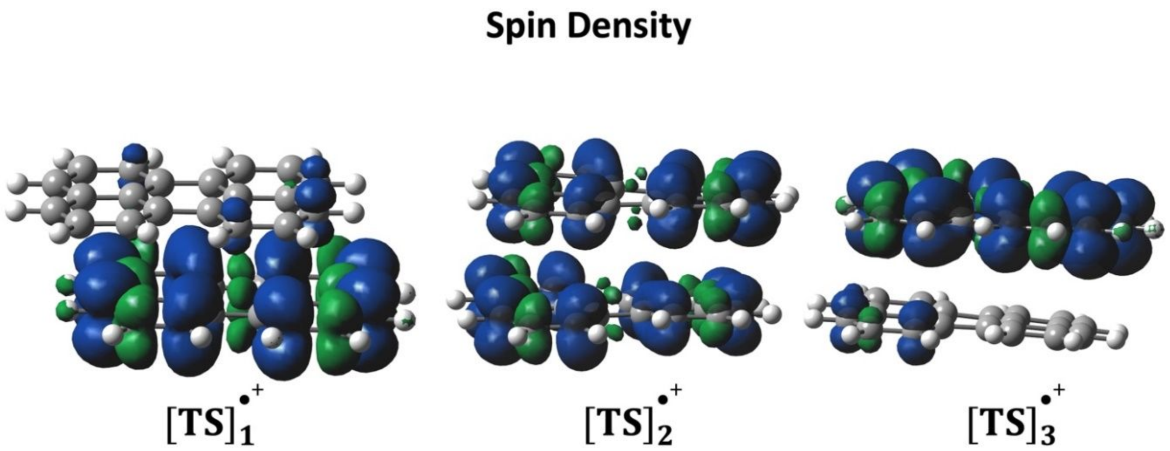

Figure S6. Spin density plot of transition states, discussed in connection with Figure 5 in the main text.

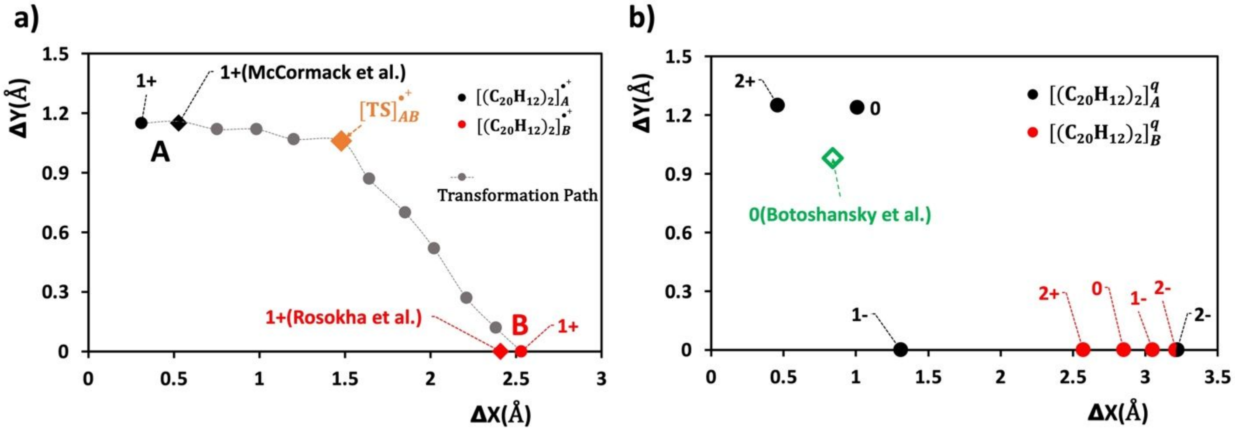

Figure S7. a) Optimized **Δ**X and **Δ**Y values of monocationic dimers and compared with those from the crystal structures by McCormack et al.[5] and Rosokha et al.[11] and after the DFT optimization for perylene dimers, with q=+1 total charge on the dimer. Grey points and the grey connecting line indicate the transformation pathway from the optimized structure of $[(C_{20}H_{12})_2]_A^{\bullet +}$ (A) to that of $[(C_{20}H_{12})_2]_B^{\bullet +}$ (B) through the transition structure, $[TS]_{AB}^{\bullet +}$. b) Optimized **Δ**X and **Δ**Y values for perylene dimers possessing total charges other than +1. The black points correspond to optimizations initiated from $[(C_{20}H_{12})_2]_A^q$ whereas the red points represent optimized structures initiated from $[(C_{20}H_{12})_2]_B^q$. The green markers represent the XRD structure of neutral perylene dimers (CSD refcode PERLEN05) by Botoshansky et al.[24]

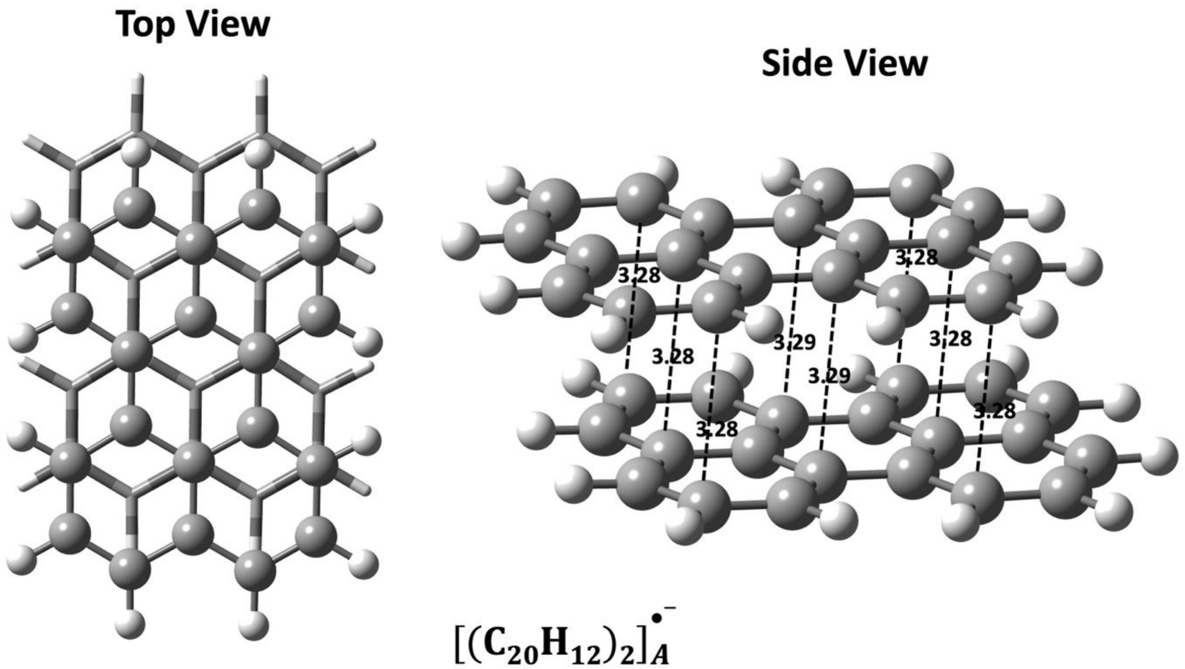

$[(C_{20}H_{12})_2]_A^{\bullet-}$

Figure S8. Optimized molecular structure of $[(C_{20}H_{12})_2]_A^{\bullet-}$ and emphasizes eight short contacts, (in Å).

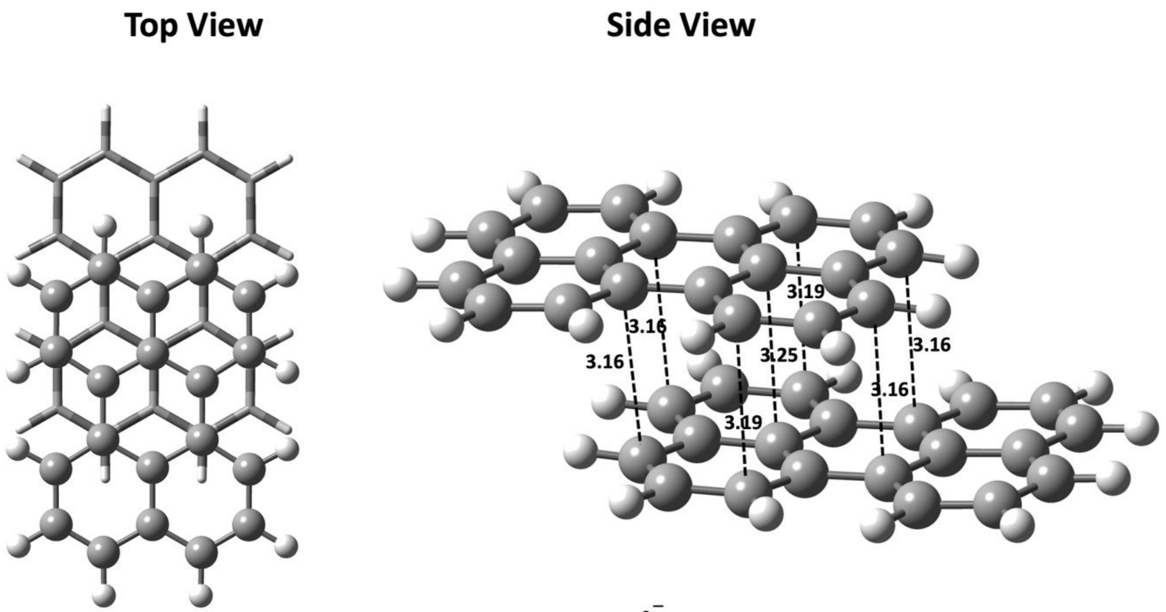

$[(C_{20}H_{12})_2]_B^{\bullet-}$

Figure S9. Optimized structure of $[(C_{20}H_{12})_2]_B^{\bullet-}$ with seven short contacts measured (in Å). (Top and side views.)

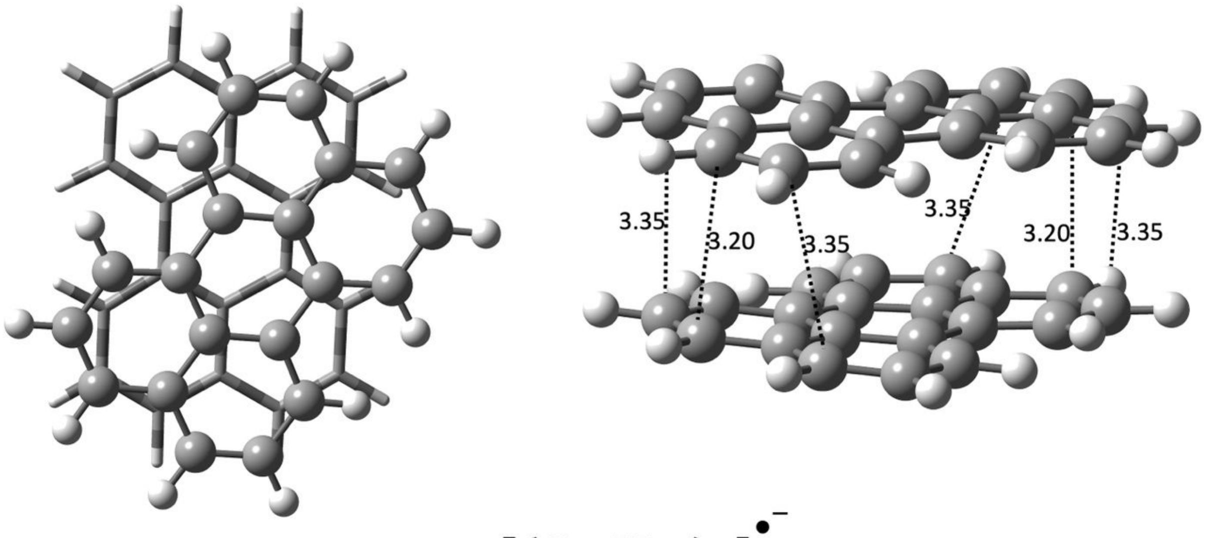

$[(C_{20}H_{12})_2]_C^{\bullet-}$

Figure S10. Optimized structure of $[(C_{20}H_{12})_2]_C^{\bullet-}$ with seven short contacts (in Å).

Spin Density

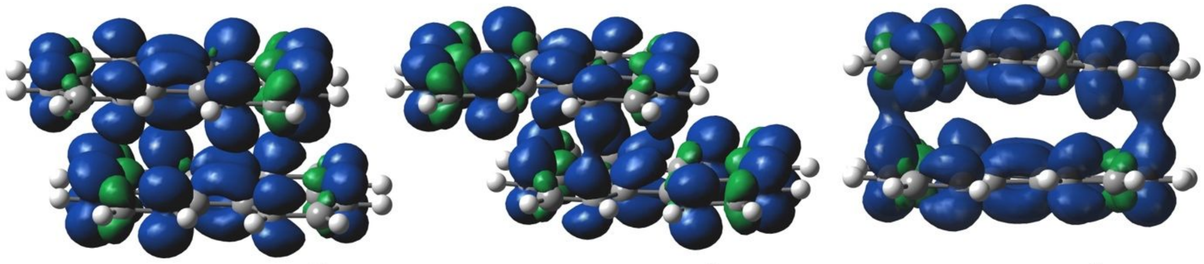

$[(C_{20}H_{12})_2]_A^{\bullet-}$ $[(C_{20}H_{12})_2]_B^{\bullet-}$ $[(C_{20}H_{12})_2]_C^{\bullet-}$

Figure S11. Spin density plot of $[(C_{20}H_{12})_2]_A^{\bullet-}$, $[(C_{20}H_{12})_2]_B^{\bullet-}$ and $[(C_{20}H_{12})_2]_C^{\bullet-}$ showing a large degree of spin delocalization within the dimer.

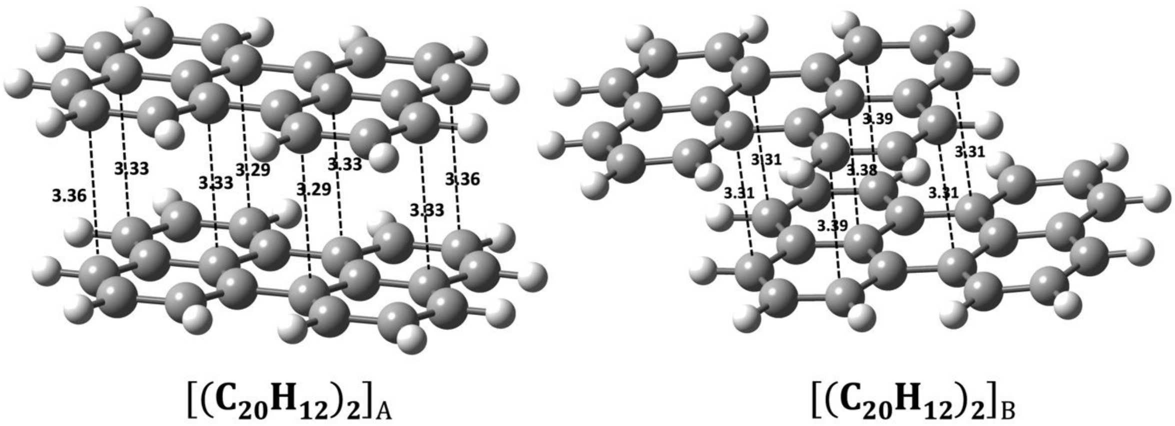

Figure S12. Optimized structure of neutral $[(C_{20}H_{12})_2]_A$ and $[(C_{20}H_{12})_2]_B$ dimers with the relevant short contacts (in Å).

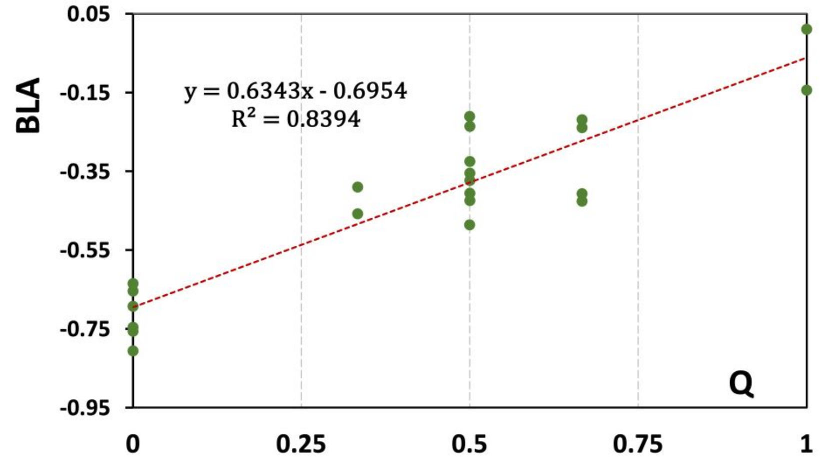

Figure S13. Correlation between assigned charges (Q) and experimentally determined BLA from the literature, as listed in Table 3. Only structures with an R-factor of less than 4% are included in this graph. The charge assignment of ¼ and ¾ in ECINAF (2($C_{20}H_{12}^+$), 3($C_{20}H_{12}$), $Mo_6O_{19}^{2-}$) has been replaced by a charge assignment of ½ and ½ compared to the data in Figure 9 and Table 3.

Charge effects on the number of unpaired electrons

An alternative assessment of the strength of pancake bonding in charged perylene dimers was considering for explaining the reduced interaction energy in the q=-1 dimers compared to the q=+1 dimers based on the fractional occupation number weighted electron density (FOD) values.[25] These were computed using the ORCA software package[26] with B3LYP/def2-TZVP. Computations were based on the geometries reported at the end of this ESI document. There is a good correlation between the N_FOD index and other measures of the number of unpaired electrons.[27]

A-type

N_FOD = 2.235099 [A(+1) dimer]
N_FOD = 2.372481 [A(-1)-dime]

B-type

N_FOD = 2.212000 [B(+1) dimer]
N_FOD = 2.366399 [B(-1) dimer]

C-type

N_FOD = 2.226899 [C(+1) dimer]
N_FOD = 2.463819 [C(-1) dimer]

There is a systematic increase of the N_FOD values, measuring the number of unpaired electrons for the q=-1 dimers compared to the q=+1 dimers. An increase in the number of unpaired electrons implies a weaker pancake bonding, and this is indeed in line with the computed pancake bond strengths. It is difficult, however, to translate the number of unpaired electrons into a strength of interaction, and for this reason, the FOD based observations should be considered only as a qualitative trend.

UV-vis spectrum prediction

The UV-Vis spectra were generated through time-dependent density functional theory (TD-DFT) calculations, employing the (U)M05-2X/6-311G(d) level of theory including 50 states.

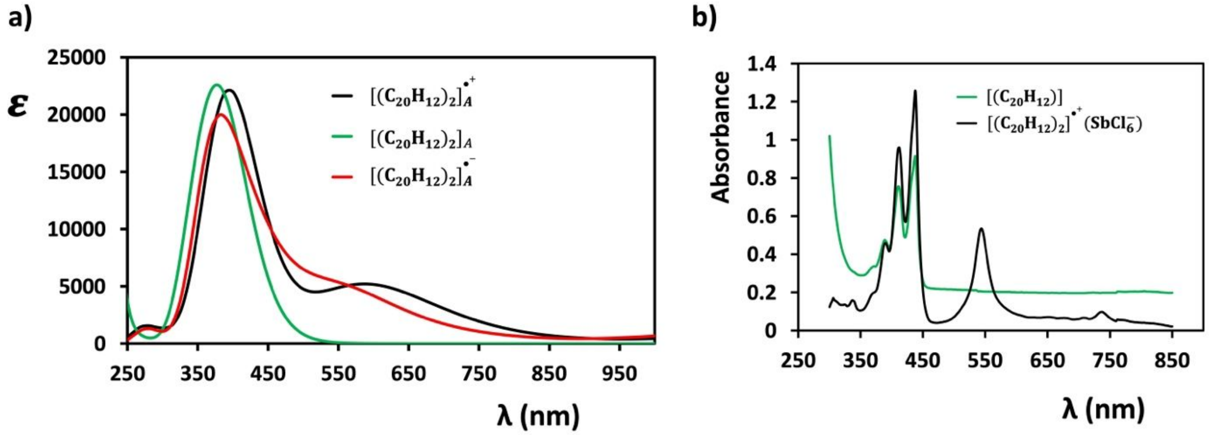

Figure S14. a) Computed UV-vis spectra of a perylene dimer. The total charge, q, of the dimer varies from +1 to -1. Spectra correspond to the minimized structure for each charged dimer, $[(C_{20}H_{12})_2]_A^{\bullet q}$. Lines were broadened by 0.333 eV. b) UV–vis absorption spectra of crystals of $[(C_{20}H_{12})_2]^{\bullet+}(SbCl_6)^-$ (Ref: XIWQON) and neat perylene dissolved in DCM, respectively. Figure S14b is adapted with permission from ref 5. Copyright [2023][ACS]

Periodic boundary condition computations

In order to further validate our dimer-based modeling, at the suggestion of a reviewer, we performed periodic boundary condition computations. Such full computations for crystals are very expensive in terms of computational resources when the unit cells are as large as many of the perylene structures analyzed in this work. However, a few examples are presented below complementing the previously presented results.

For XIWQON (Formula: $[(C_{20}H_{12})_2]^{\bullet+}(SbCl_6)^-$), the crystal serves as an ideal comparison to the isolated dimer modeling. The unit cell contains an isolated perylene dimer with a total formal charge (q) of +1. Through periodic boundary condition calculations[28] using the PBE functional with kinetic energy cut-off of 70 Ry and norm-conserving pseudopotentials, we have verified that the HOMO-1 orbital exhibits intermolecular orbital overlap, playing a key role in the formation of the pancake bond, (see Figure S15) akin to the isolated dimer presented in the manuscript. Additionally, we conducted Bader charge computations[29] for the perylenes, reinforcing our conclusion of an equal charge share between the two perylenes in the dimer.

Conversely, the unit cell for ECINEJ is more intricate, comprising five perylenes and one metal-oxo cluster as the counter anion. Direct comparison with the isolated dimer is challenging. Nevertheless, the orbitals exhibit distinct pancake bonding in the occupied orbital. Specifically, HOMO-2, HOMO-3, and HOMO-4 orbitals clearly reveal characteristic intermolecular overlaps of different types of pancake interactions (Type A, C) within the unit cell (see Figure S16). Bader charge computations for each perylene indicate that two perylenes carry approximately +0.75e each, two perylenes possess approximately +0.5e charge each, and one perylene has approximately +0.2e charge (Figure S17). These charge distributions align well with our assigned values as shown in the small tables below. Given the highly approximate nature of any charge definition, the agreement is excellent for both crystals between the quantum mechanically computed Bader charges, and the charges assigned in the main text based on the BLA-charge correlation represented in Figure 2. It is clear that in a crystal computation some of the charges are assigned to the intermolecular space that is assigned fully to the molecules and counterions in the molecular model.

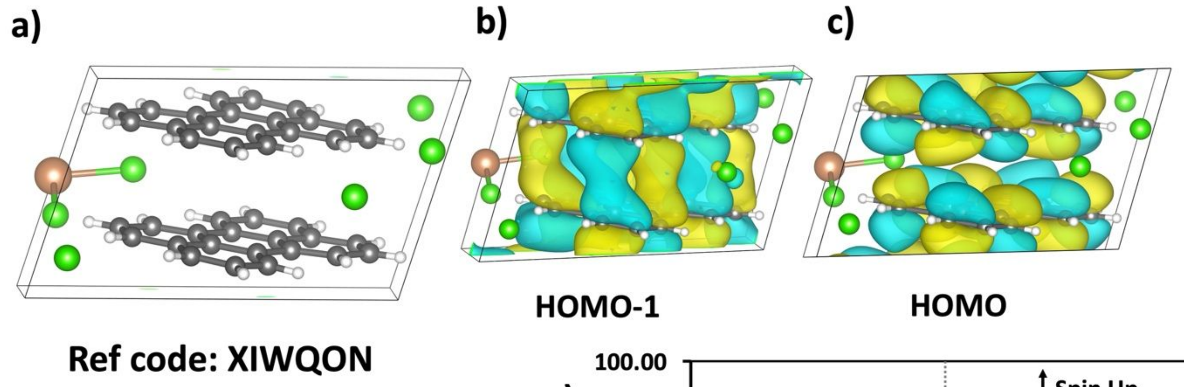

d)

Molecular Fragment	Bader Charge	Assigned Charge
**Perylene 1**	+0.45	+0.50
**Perylene 2**	+0.45	+0.50
**Counter anion**	-0.90	-1.00
**Total Charge**	0.00	0.00

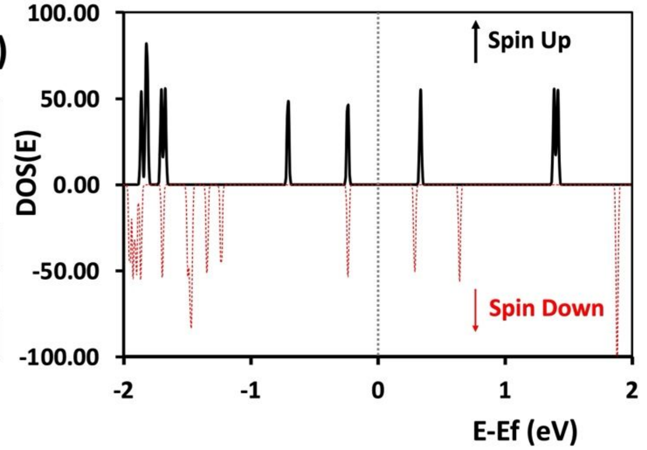

Figure S15. a) Unit Cell of XIWQON ($(C_{20}H_{12})_2]^{\bullet+}(SbCl_6)^{-})$), b) HOMO-1 orbital, c) HOMO orbital, d) Bader charge distribution, e) Density of States (DOS).

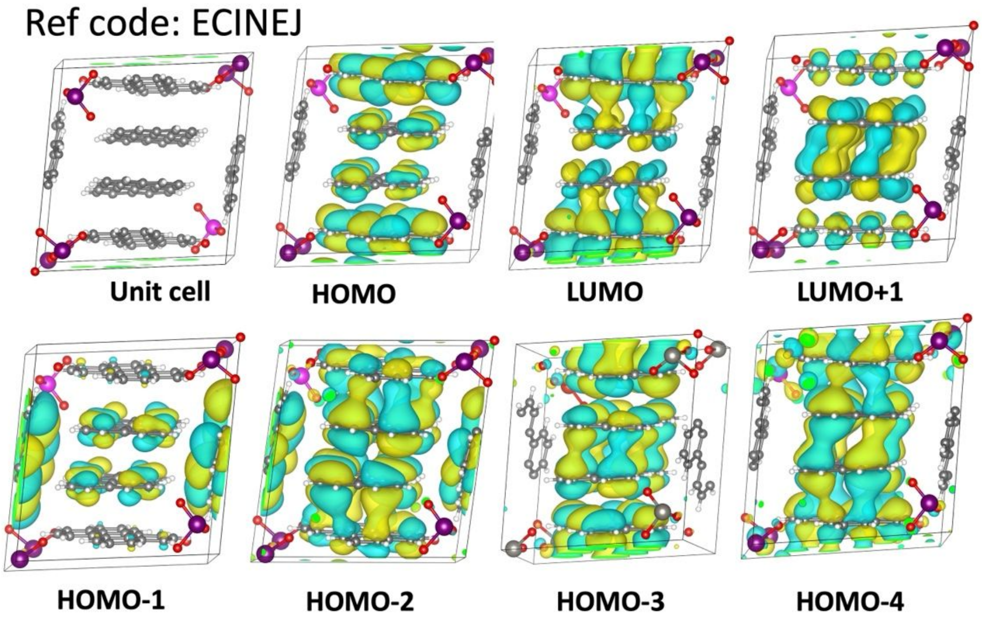

Figure S16. Unit cell of ECINEJ ($3(C_{20}H_{12}^{+})$, $2(C_{20}H_{12})$, $O_{19}VW_5^{3-}$) and a few relevant orbitals at k=0 showing pancake bonds.

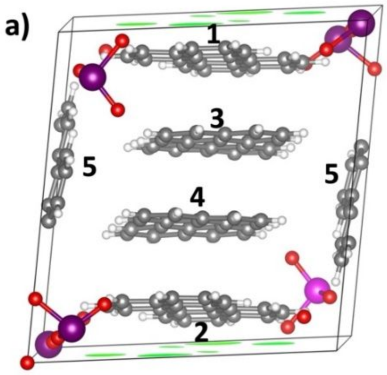

Molecular Fragment	Bader Charge	Assigned Charge
Perylene 1	+0.76	+1.0
Perylene 2	+0.73	+1.0
Perylene 3	+0.53	+0.5
Perylene 4	+0.51	+0.5
Perylene 5	+0.23	0.0
Counter anion	-2.76	-3.0
Total Charge	0.00	0.00

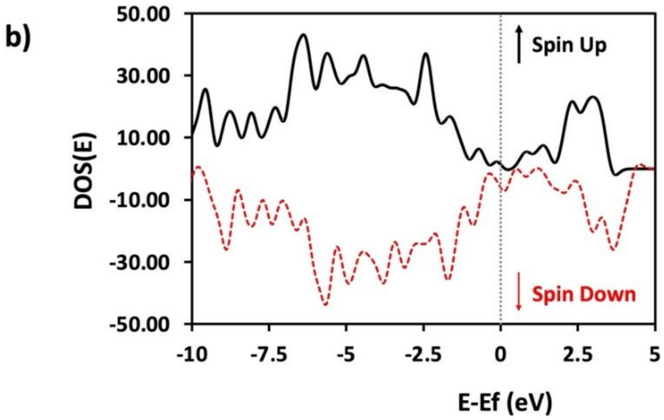

Figure S17. a) Unit cell of ECINEJ (3($C_{20}H_{12}^{+}$), 2($C_{20}H_{12}$), $O_{19}VW_5^{3-}$) and Bader charge distribution, b) Density of states (DOS) of the system.

Assessment of aromaticity by HOMA

As suggested by a reviewer, we also calculated the Harmonic Oscillator Measure of Aromaticity (HOMA) index to assess aromaticity changes in the charged dimers.[30, 31] As shown in Table S7, the HOMA index is slightly higher in the mono-cationic dimers, contributing to their enhanced stability. We observe the same trend such that pancake bonding enhances the overall aromaticity as measured by the HOMA index.[30]

Table S8: Harmonic Oscillator Measure of Aromaticity (HOMA) index of dimers at various charges.

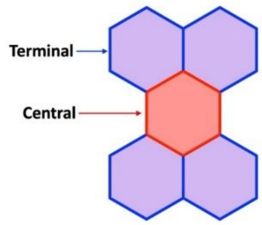

Harmonic Oscillator Measure of Aromaticity (HOMA) index			
Optimized Dimer	Central ring (Average)	Terminal ring (Average)	$\overline{HOMA}$
$[C_{20}H_{12}]_A^{+1}$	0.337	0.862	0.76
$[C_{20}H_{12}]_A$	0.089	0.825	0.68
$[C_{20}H_{12}]_A^{-1}$	0.281	0.827	0.72
$[C_{20}H_{12}]_B^{+1}$	0.359	0.860	0.76
$[C_{20}H_{12}]_B$	0.108	0.832	0.68
$[C_{20}H_{12}]_B^{-1}$	0.290	0.823	0.72
$[C_{20}H_{12}]_C^{+1}$	0.362	0.865	0.76
$[C_{20}H_{12}]_C$	0.094	0.825	0.68
$[C_{20}H_{12}]_C^{-1}$	0.288	0.829	0.72

QTAIM analysis

In response to a reviewers recommendations, we have undertaken QTAIM analysis[32] for A-type dimers with total charges of +1, 0, and -1 to discern any trends. These are listed in Tables S9, S10, and S11 using the Multiwfn code.[33] Due to the multicenter nature of pancake bonding, each individual bond listed in the table contributes to the overall interaction. Each contribution is small as expected, given the relatively large (compared to covalent bonds) distances and small electron densities. However, it is worth noting that QTAIM analysis does not contribute significantly to distinguishing the nature of the pancake bonding interaction in our systems.

Table S9: QTAIM analysis[a] of $[C_{20}H_{12}]_A^{+1}$

QTAIM	*PCB*	$\nabla^2\rho(r)$ (a.u)	$G(r)$ (a.u)	$V(r)$ (a.u)	$V(r)/2$ kcal/mol	$\frac{\lvert V(r)\rvert}{G(r)}$	$H(r)$ (a.u)	$\rho(r)$ (a.u.)	$\frac{H(r)}{\rho(r)}$
$[C_{20}H_{12}]_A^{+1}$	C49---C31	0.017322	0.003654	-0.002979	-0.93	0.8152	0.000676	0.006939	0.0974
	C33---C23	0.020505	0.0043490	-0.003572	-1.12	0.8213	0.000777	0.007978	0.0973
	C35---C4	0.020781	0.0044005	-0.003606	-1.13	0.8194	0.000795	0.007396	0.1075
	C43---C10	0.018490	0.003873	-0.003124	-0.98	0.8066	0.000749	0.006926	0.1081
	C42---C11	0.018483	0.003872	-0.003122	-0.98	0.8063	0.000749	0.006924	0.1082
	C36---C3	0.020784	0.004401	-0.003606	-1.13	0.8193	0.000795	0.007397	0.1075
	C55---C1	0.020476	0.0043426	-0.003566	-1.12	0.8211	0.000776	0.007969	0.0974
	C63---C17	0.017324	0.003655	-0.002979	-0.93	0.8150	0.000676	0.006940	0.0974

[a]Notes for Tables S9, S10, and S11:
BCP: Bond Critical Point; PCB(BCP): BCP of Pancake Bond ; ρ(r): Density of all electrons.
∇^2ρ(r): Laplacian of electron density; G(r): Lagrangian kinetic energy; V(r):Potential energy density; H(r): Energy density.

Table S10: QTAIM analysis of $[C_{20}H_{12}]_A^{-1}$

QTAIM	PCB	$\nabla^2\rho(r)$ (a.u.)	G(r) (a.u.)	V(r) (a.u.)	$V(r)/2$ $kcal/mol$	$\frac{\lvert V(r)\rvert}{G(r)}$	H(r) (a.u.)	$\rho(r)$ (a.u.)	$\frac{H(r)}{\rho(r)}$
$[C_{20}H_{12}]_A^{-1}$	C59---C13	0.020478	0.004457	-0.003795	-1.19	0.8514	0.000662	0.008013	0.0827
	C39---C6	0.021397	0.004514	-0.003679	-1.15	0.8150	0.000835	0.007260	0.1150
	C57---C8	0.020477	0.004457	-0.003795	-1.19	0.8515	0.000662	0.008013	0.0826
	C34---C5	0.020576	0.004414	-0.003685	-1.16	0.8347	0.000730	0.007562	0.0965
	C37---C2	0.020586	0.004417	-0.00369	-1.16	0.8347	0.000730	0.007568	0.0964
	C40---C25	0.020407	0.004441	-0.003781	-1.19	0.8513	0.000661	0.007987	0.0827
	C38---C7	0.021390	0.004513	-0.003678	-1.15	0.8150	0.000835	0.007258	0.1150
	C45---C27	0.020570	0.004478	-0.003813	-1.20	0.8516	0.000665	0.008044	0.0826

Table S11: QTAIM analysis of $[C_{20}H_{12}]_A^0$

QTAIM	PCB (BCP)	$\nabla^2\rho(r)$ (a.u.)	G(r) (a.u.)	V(r) (a.u.)	$V(r)/2$ $kcal/mol$	$\frac{\lvert V(r)\rvert}{G(r)}$	H(r) (a.u.)	$\rho(r)$ (a.u.)	$\frac{H(r)}{\rho(r)}$
$[C_{20}H_{12}]_A$	C49---C31	0.017767	0.003759	-0.003076	-0.96	0.8183	0.000683	0.006675	0.1023
	C33---C23	0.020442	0.004322	-0.003533	-1.11	0.8175	0.000788	0.007295	0.1080
	C35---C4	0.019463	0.004113	-0.003360	-1.05	0.8170	0.000753	0.006972	0.1079
	C43---C10	0.019204	0.004043	-0.003285	-1.03	0.8125	0.000758	0.007071	0.1072
	C42---C11	0.019217	0.004046	-0.003288	-1.03	0.8127	0.000758	0.007074	0.1071
	C36---C3	0.019461	0.004113	-0.003360	-1.05	0.8170	0.000752	0.006969	0.1079
	C55---C1	0.020483	0.004331	-0.003541	-1.11	0.8176	0.000790	0.007304	0.1081
	C63---C17	0.017772	0.003761	-0.003079	-0.96	0.8186	0.000682	0.006680	0.1021

Local vibrational mode force constant analysis

Following the reviewer's recommendation, we employed a local vibrational mode force constant analysis using the LMode-nano code[34, 35] to calculate Local Stretching Force Constants (LSFC). In this analysis, we computed the local force constants for all C···C short contacts of $[C_{20}H_{12}]_A^{+1/-1}$ and $[C_{20}H_{12}]_B^{+1/-1}$, as detailed in Table S12 below. The table reveals that the C···C force constants for negatively charged dimers are slightly larger compared to positive dimers. Consequently, the higher interaction energies in mono-cationic dimers stem from other contributing factors.

Table S12: Local mode force constants, $k_n^a(CC)$ of CC bonds involved in pancake bonding.

CC Short Contacts	$k_n^a(CC)$ [mdyn/Å]<br>$k_n^a(CC)$ =Local Mode Force Constants between Two Atoms			
	$[C_{20}H_{12}]_A^{+1}$	$[C_{20}H_{12}]_A^{-1}$	$[C_{20}H_{12}]_B^{+1}$	$[C_{20}H_{12}]_B^{-1}$
$(C\cdots C)_1$	0.192	0.201	0.264	0.333
$(C\cdots C)_2$	0.192	0.201	0.126	0.159
$(C\cdots C)_3$	0.116	0.179	0.126	0.159
$(C\cdots C)_4$	0.116	0.179	0.126	0.159
$(C\cdots C)_5$	0.091	0.071	0.126	0.158
$(C\cdots C)_6$	0.091	0.07	0.07	0.126
$(C\cdots C)_7$	0.076	0.071	0.07	0.125
$(C\cdots C)_8$	0.076	0.071	--	--

Optimized Coordinates (in Å) are provided as a separate XYZ file.

References:

(1) Zhao, Y.; Truhlar, D. G. Density functionals with broad applicability in chemistry. *Acc. Chem. Res.* **2008**, *41* (2), 157-167.
(2) Mou, Z.; Tian, Y.-H.; Kertesz, M. Validation of density functionals for pancake-bonded π-dimers; dispersion is not enough. *Phys.l Chem. Chem. Phys.* **2017**, *19* (36), 24761-24768.
(3) Szalay, P. G.; Bartlett, R. J. Multi-reference averaged quadratic coupled-cluster method: a size-extensive modification of multi-reference CI. *Chem. Phys. Lett.* **1993**, *214* (5), 481-488.
(4) Grimme, S.; Antony, J.; Ehrlich, S.; Krieg, H. A consistent and accurate ab initio parametrization of density functional dispersion correction (DFT-D) for the 94 elements H-Pu. *J. Chem. Phys.* **2010**, *132* (15).
(5) McCormack, M. E.; Bhattacharjee, R.; Jervis, H.; Wei, Z.; Kertesz, M.; Petrukhina, M. A. Stabilizing Cationic Perylene Dimers through Pancake Bonding and Equal Charge Share. *Cryst. Growth Des.* **2023**, *23* (10), 7496–7503. DOI: 10.1021/acs.cgd.3c00912.
(6) Coronado, E.; Galán-Mascarós, J. R.; Giménez-Saiz, C.; Gómez-García, C. J.; Martínez-Ferrero, E.; Almeida, M.; Lopes, E. B.; Capelli, S. C.; Llusar, R. M. New conducting radical salts based upon Keggin-type polyoxometalates and perylene. *J. Mater. Chem.* **2004**, *14* (12), 1867-1872.

(7) Jeannin, O.; Fourmigué, M. Perylene salts of unsymmetrical nickel and gold dithiolene complexes with 3∶ 2 stoichiometry: conformational polymorphism and strong antiferromagnetic interactions. *New J. Chem.* **2006**, *30* (12), 1774-1781.
(8) Santos, I. C.; Ayllon, J. A.; Henriques, R. T.; Almeida, M.; Alcácer, L.; Duarte, M. T. A New Perylene Salt: Diperylenium (1+) Bis [quinoxaline-2, 3-dithiolato (2–)-S, S'] cuprate (III). *Acta. Cryst.* **1997**, *C53* (12), 1768-1770.
(9) Endres, H.; Keller, H. J.; Müller, B.; Schweitzer, D. Electrocrystallization and structures of perylene radical salts: hexaperylene perchlorate,$(C_{20}H_{12})$ 6+ ClO_4^-, triperylene perchlorate,$(C_{20}H_{12})$ 3+ ClO_4^-, and diperylene hexafluorophosphate–tetrahydrofuran (3/2),$(C_{20}H_{12})$ 2+ PF_6^-. 2/3C_4H_8O. *Acta. Cryst.* **1985**, *41* (4), 607-613.
(10) Clemente-León, M.; Coronado, E.; Giménez-Saiz, C.; Gómez-García, C. J.; Martínez-Ferrero, E.; Almeida, M.; Lopes, E. B. Organic/inorganic molecular conductors based upon perylene and Lindquist-type polyoxometalates. *J. Mater. Chem.* **2001**, *11* (9), 2176-2180.
(11) Rosokha, S. V.; Stern, C. L.; Ritzert, J. T. π-Bonded molecular wires: self-assembly of mixed-valence cation-radical stacks within the nanochannels formed by inert tetrakis [3, 5-bis (trifluoromethyl) phenyl] borate anions. *CrystEngComm* **2013**, *15* (48), 10638-10647.
(12) Domingos, A.; Henriques, R.; Gama, V.; Almeida, M.; Vieira, A. L.; Alcácer, L. Crystalline structure/transport properties relationship in the $(perylene)_2M(mnt)_2$ family M= Au, Pd, Pt, Ni. *Synth. Met.* **1988**, *27* (3-4), 411-416.
(13) Matos, M.; Bonfait, G.; Santos, I. C.; Afonso, M. L.; Henriques, R. T.; Almeida, M. The solid solutions $(Per)_2[Pt_xAu$ (1− x)$(mnt)_2]$; Alloying para-and diamagnetic anions in two-chain compounds. *Magnetochemistry* **2017**, *3* (2), 22.
(14) Gama, V.; Henriques, R. T.; Bonfait, G.; Pereira, L. C.; Waerenborgh, J. C.; Santos, I. C.; Duarte, M. T.; Cabral, J. M.; Almeida, M. Low-dimensional molecular metals bis (maleonitriledithiolato) bis (perylene) metal, metal= iron and cobalt. *Inorg. Chem.* **1992**, *31* (12), 2598-2604.
(15) Almeida, M.; Gama, V.; Santos, I. C.; Graf, D.; Brooks, J. S. Counterion dimerisation effects in the two-chain compound $(Per)_2[Co\ (mnt)_2]$: structure and anomalous pressure dependence of the electrical transport properties. *CrystEngComm* **2009**, *11* (6), 1103-1108.
(16) Shibaeva, R.; Kaminskii, V.; Yagubskii, E. Crystal structures of organic metals and superconductors of (BEDT-TTP)-I system. *Mol. Cryst. Liq. Cryst.* **1985**, *119* (1), 361-373.
(17) Ayllón, J. A.; Santos, I. C.; Henriques, R. T.; Almeida, M.; Lopes, E. B.; Morgado, J.; Alcácer, L.; Veiros, L. F.; Duarte, M. T. Perylene salts with tetrahalogenoferrate (III) anions. Synthesis, crystal structure of $[(C_{20}\ H_{12})_3][FeCl_4]$ and characterisation. *J. Chem. Soc., Dalton Trans.* **1995**, (21), 3543-3549.
(18) Kuhs, W.; Mattern, G.; Brütting, W.; Dragan, H.; Burggraf, M.; Pilawa, B.; Dormann, E. Electrocrystallization, crystal structure and physical properties of hexaperylene hexafluorophosphate,$(C_{20}H_{12})_6.^+.PF_6^-$. *Acta Cryst.* **1994**, *B50* (6), 741-746.
(19) Keller, H. J.; Nöthe, D.; Pritzkow, H.; Wehe, D.; Werner, M.; Koch, P.; Schweitzer, D. Electrochemically generated peryleniumyl-hexafluorophosphate and hexafluoroarsenate: new one-dimensional metals. *Mol.Cryst.Liq.Cryst.* **1980**, *62* (3-4), 181-199.
(20) Gama, V.; Henriques, R. T.; Almeida, M.; Veiros, L.; Calhorda, M.; Meetsma, A.; de Boer, J. L. A novel trinuclear cobalt complex: crystal and electronic structure of perylene bis (maleonitriledithiolato) cobaltate $(Per)_4[Co\ (mnt)_2]_3$. *Inorg. Chem.* **1993**, *32* (17), 3705-3711.

(21) Bock, H.; Näther, C.; Havlas, Z.; John, A.; Arad, C. Ether-Solvated Sodium Ions in Salts Containing π-Hydrocarbon Anions: Crystallization, Structures, and Semiempirical Solvation Energies. *Angew. Chem. Int. Ed.* **1994**, *33* (8), 875-878.
(22) Näther, C.; Bock, H.; Havlas, Z.; Hauck, T. Solvent-shared and solvent-separated ion multiples of perylene radical anions and dianions: an exemplary case of alkali metal cation solvation. *Organometallics* **1998**, *17* (21), 4707-4715.
(23) Castillo, M.; Metta-Magaña, A. J.; Fortier, S. Isolation of gravimetrically quantifiable alkali metal arenides using 18-crown-6. *New J. Chem.* **2016**, *40* (3), 1923-1926.
(24) Botoshansky, M.; Herbstein, F. H.; Kapon, M. Towards a complete description of a polymorphic crystal: The example of perylene: Redetermination of the structures of the (Z= 2 and 4) polymorphs. *Helv. Chim. Acta.* **2003**, *86* (4), 1113-1128.
(25) Grimme, S.; Hansen, A. A practicable real-space measure and visualization of static electron-correlation effects. *Angew. Chem. Int. Ed.* **2015**, *54* (42), 12308-12313.
(26) Neese, F. The ORCA program system. *Wiley Interdisciplinary Reviews: Computational Molecular Science* **2012**, *2* (1), 73-78.
(27) Nieman, R.; Carvalho, J. R.; Jayee, B.; Hansen, A.; Aquino, A. J.; Kertesz, M.; Lischka, H. Polyradical character assessment using multireference calculations and comparison with density-functional derived fractional occupation number weighted density analysis. *Phys. Chem. Chem. Phys.* **2023**, *25* (40), 27380-27393.
(28) Giannozzi, P.; Baroni, S.; Bonini, N.; Calandra, M.; Car, R.; Cavazzoni, C.; Ceresoli, D.; Chiarotti, G. L.; Cococcioni, M.; Dabo, I. QUANTUM ESPRESSO: a modular and open-source software project for quantum simulations of materials. *J. Phys. Condens. Matter* **2009**, *21* (39), 395502.
(29) Henkelman, G.; Arnaldsson, A.; Jónsson, H. A fast and robust algorithm for Bader decomposition of charge density. *Comput. Mater. Sci.* **2006**, *36* (3), 354-360.
(30) Krygowski, T. M. Crystallographic studies of inter-and intramolecular interactions reflected in aromatic character of. pi.-electron systems. *J. Chem. Inf. Comput. Sci.* **1993**, *33* (1), 70-78.
(31) Kruszewski, J.; Krygowski, T. Definition of aromaticity basing on the harmonic oscillator model. *Tetrahedron Lett.* **1972**, *13* (36), 3839-3842.
(32) Bader, R. F. A quantum theory of molecular structure and its applications. *Chem. Rev.* **1991**, *91* (5), 893-928.
(33) Lu, T.; Chen, F. Multiwfn: A multifunctional wavefunction analyzer. *J. Comput. Chem.* **2012**, *33* (5), 580-592.
(34) Delgado, A. A. A.; Humason, A.; Kraka, E. Pancake bonding seen through the eyes of spectroscopy. *Density Functional Theory—Recent Advances, New Perspectives and Applications* **2021**, 1-21.
(35) Tao, Y.; Zou, W.; Nanayakkara, S.; Kraka, E. LModeA-nano: a PyMOL plugin for calculating bond strength in solids, surfaces, and molecules via local vibrational mode analysis. *J. Chem. Theory Comput.* **2022**, *18* (3), 1821-1837.
